# A community and functional comparison of coral and reef fish assemblages between four decades of coastal urbanisation and thermal stress

**DOI:** 10.1002/ece3.8736

**Published:** 2022-03-22

**Authors:** Katie M. Cook, Hirotaka Yamagiwa, Maria Beger, Giovanni Diego Masucci, Stuart Ross, Hui Yian Theodora Lee, Rick D. Stuart‐Smith, James Davis Reimer

**Affiliations:** ^1^ School of Biology Faculty of Biological Sciences University of Leeds Leeds UK; ^2^ Molecular Invertebrate Systematics and Ecology Laboratory Graduate School of Engineering and Science University of the Ryukyus Nishihara Japan; ^3^ Centre for Biodiversity and Conservation Science School of Biological Sciences The University of Queensland Brisbane Queensland Australia; ^4^ Experimental Marine Ecology Laboratory Department of Biological Sciences National University of Singapore Singapore Singapore; ^5^ Institute for Marine and Antarctic Studies University of Tasmania Taroona Tasmania Australia; ^6^ Tropical Biosphere Research Center University of the Ryukyus Nishihara Japan

**Keywords:** coastal reefs, community turnover, functional traits, Okinawa, temporal change, Urbanization

## Abstract

1. Urbanized coral reefs experience anthropogenic disturbances caused by coastal development, pollution, and nutrient runoff, resulting in turbid, marginal conditions in which only certain species can persist. Mortality effects are exacerbated by increasingly regular thermal stress events, leading to shifts towards novel communities dominated by habitat generalists and species with low structural complexity.
2. There is limited data on the turnover processes that occur due to this convergence of anthropogenic stressors, and how novel urban ecosystems are structured both at the community and functional levels. As such, it is unclear how they will respond to future disturbance events.
3. Here, we examine the patterns of coral reef community change and determine whether ecosystem functions provided by specialist species are lost post‐disturbance. We present a comparison of community and functional trait‐based changes for scleractinian coral genera and reef fish species assemblages subject to coastal development, coastal modification, and mass bleaching between two time periods, 1975–1976 and 2018, in Nakagusuku Bay, Okinawa, Japan.
4. We observed an increase in fish habitat generalists, a dominance shift from branching to massive/sub‐massive corals and increasing site‐based coral genera richness between years. Fish and coral communities significantly reassembled, but functional trait‐based multivariate space remained constant, indicating a turnover of species with similar traits. A compression of coral habitat occurred, with shallow (<5 m) and deep (>8 m) coral genera shifting towards the mid‐depths (5–8 m).
5. We show that although reef species assemblages altered post disturbance, new communities retained similar ecosystem functions. This result could be linked to the stressors experienced by urban reefs, which reflect those that will occur at an increasing frequency globally in the near future. Yet, even after shifts to disturbed communities, these fully functioning reef systems may maintain high conservation value.

## Graphical abstract

Our study compared coral and fish communities between 1975 and 2018, in Nakagusuku Bay, Okinawa, Japan. Over 43 years of urbanization and thermal stress, coral and fish assemblages changed significantly, but functional trait space was conserved, with both taxa experiencing even turnovers across space. We observed a shift in dominant coral morphology from branching to massive, and a “depth compression” of corals, with the average depths of deep and shallow coral genera compressing towards the mid‐depths.

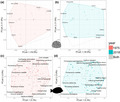

## 1 INTRODUCTION

Coral reefs are severely threatened by anthropogenic disturbances and climate change, with a significant loss of global coral cover recorded in the last few decades (Hughes, Kerry, et al., 2018). As well as losses associated with increasingly frequent and severe global mass coral bleaching events (Hughes, Anderson, et al., 2018; Sully et al., 2019), coastal urbanization threatens water quality, water fluxes, and sustainability of extractive use for nearshore coral reefs (Masucci & Reimer, 2019). Such disturbances result in reassembly of communities, with the turnover of certain species, taxa, and functional groups (Nyström et al., 2008; Stuart‐Smith et al., 2018). Disturbed communities often have reduced structural complexity, losing the capacity to maintain diversity and altering trophic structure (Cruz et al., 2018). The loss of microhabitats can cause communities to become homogenized and dominated by habitat generalists (Stuart‐Smith et al., 2021; Wilson et al., 2008). The loss of complexity is especially pronounced on urban reefs (Januchowski‐Hartley et al., 2020), but its effects on trait communities and functioning remain poorly known.

As the human population increases, coastal zones are experiencing rapid rates of urbanization, resulting in land reclamation, artificial rocky habitats for flood prevention and the building of harbors and piers (Heery et al., 2018). The marine environment can further be altered by increased sedimentation, nutrient runoff, and the introduction of toxic heavy metals and organic contaminants (Pollock et al., 2014). These processes threaten reef building corals by increasing turbidity, disease prevalence, and reducing coral reproduction (Browne, 2012). Yet, scleractinian coral reefs can still be found adjacent to established tropical and subtropical urban areas (Hongo & Yamano, 2013). These turbid urban reefs differ in composition to offshore reefs, but there is limited data to understand the turnover processes that occur due to urbanization (Heery et al., 2018). Furthermore, it is unknown if these ecosystems are structurally and functionally unique, and how they will respond to further environmental stress (Heery et al., 2018). It has been suggested that species persisting in marginal conditions may be preadapted to be resilient to further stressors such as bleaching events (Guest et al., 2016; Sofonia & Anthony, 2008).

Mass bleaching events caused by prolonged periods of thermal stress have occurred with increasing frequency in the last four decades (Hughes, Kerry, et al., 2018), with differential responses to thermal stress exhibited by coral genera (Kim et al., 2019). In Japan, live coral cover was reduced by 85% in some areas due to severe bleaching events that started in 1998, mostly killing branching coral morphologies such as *Acropora* sp. (Loya et al., 2001). Post‐bleaching, Japanese coral communities have been dominated by massive (boulder) and encrusting morphologies, and thermally susceptible branched corals have almost completely disappeared (Loya et al., 2001). However, branching and plating colonies experienced differing degrees of bleaching mortality, suggesting factors other than coral morphology also affect survival. For example, corals found across a large depth range are likely to be habitat generalists, pre‐adapted to survive under a range of thermal conditions (Bongaerts & Smith, 2019; Chow et al., 2019). Shallow specialists thrive under high light levels, high wave energy, and low sediment deposition, but if disturbance alters these conditions, survival is less likely (Chow et al., 2019). Deeper corals may be able to repopulate shallow areas after mortality (Holstein et al., 2015; Smith et al., 2014), particularly if they have a high dispersal capacity (Graham et al., 2008).

Corals that survive disturbance events and those that repopulate degraded reefs may have similar functional traits (Chow et al., 2019). Traits can determine species abiotic tolerances, as well as biotic interactions such as competition, feeding, and predation (Hébert et al., 2015). Thus, they are linked to ecosystem functioning, which considers how interactions between the biological assemblages of the system determine critical processes such as energy flow and community properties such as resilience (Reiss et al., 2009). If disturbances favor specific traits, the mortality of whole groups of species with different unique traits could occur, reducing the capacity of the ecosystem to function (Siwicka et al., 2020). For example, on tropical reefs, zooxanthellate corals are the habitat builders, and the structural complexity of the reef can determine the abundance and diversity of reef associated species (Darling et al., 2017). Corals with complex morphologies provide shelter and nursery habitats for reef fish (Hamilton et al., 2017). If all branching corals are lost, these fish may also be lost from the reef.

Diverse fish communities perform a multitude of functions, and their resilience to both fishing and coral habitat degradation has been linked to the functional traits of the component species (Streit et al., 2019). For example, herbivorous fish help prevent phase shifts from coral to algal dominated ecosystems and are critical in maintaining a functioning reef community (Edwards et al., 2014). Furthermore, they provide prey to larger fish species that provide top down predation, keeping the ecosystem in equilibrium (Valdivia et al., 2017). A healthy, diverse reef system supports fish species with a wide range of specialized functional niches (Mouillot et al., 2014). However, similar to corals, shifts to more generalized fish communities have been observed in degraded systems (Richardson et al., 2018; Stuart‐Smith et al., 2021). This indicates reduced ecosystem functioning, feeding back to further coral losses (Richardson et al., 2018). Thus, the resilience of coral reef ecosystems to disturbances is not only related to the corals themselves but the interactions among species and taxa. Therefore, it is also important to understand how fish communities and their functions change with disturbance to help understand future community resilience and ecosystem change.

The coastline and reefs of Okinawa Island, Japan, present a good model system to study the combined impacts of urbanization (Masucci & Reimer, 2019) and climate stress (Hongo & Yamano, 2013) on coastal coral reefs. Okinawa's coasts have supported ports and naval bases since World War Two, creating extensive disturbances particularly in the bays on the southeast coast. In the post‐war period, after Okinawa reverted from US occupation to Japan in 1972, the Japanese government invested heavily in Okinawa's development, supporting the farming and manufacturing industries, large‐scale construction projects, and tourism. Development resulted in a population boom, and from 1955 to 1990, the population of Okinawa prefecture increased by 53% from 800,000 to 1.22 million people (Kuwahara, 2012; Tada, 2016). Currently, Okinawa Prefecture has a population of 1.45 million and attracts over 10 million tourists per year (Aizawa et al., 2014; Hifumi et al., 2020). The economic development from the 1970s led to rapid coastal development, with an acceleration in dredging, landfilling, and terrestrial runoff (Japan Coral Reef Society, 2004; Masucci & Reimer, 2019; Omori, 2011). This has resulted in the creation of turbid urbanized reefs with high levels of suspended sediments and reduced water transparency (Hongo & Yamano, 2013). However, the long‐term community changes of these urban reefs are not well known.

Here we examine the changes in community and functional composition of corals and reef fish in Nakagusuku Bay, Okinawa, Japan, between two time periods, 1975–1976 and 2018. These 43 years span the majority of the years of Okinawa's extensive coastal development, as well as four global mass coral bleaching events. We determine whether anthropogenic disturbances have resulted in the reassembly of coral and fish communities. To understand the reassembly processes we explored: (1) the change in coral genera coverage and the average depths of coral genera occurrence, (2) the change in coral and fish community composition and richness, (3) fish community homogenization, and (4) changes in functional trait‐based community space. Under ongoing climate change, and with the ongoing increase in the global human population, turbid urbanized reefs may become increasingly dominant. Quantifying changes that occur in these reef communities is critical to understand how currently more “pristine” reefs may look in the future and how urbanized reefs may continue to change.

## 2 METHODS

### 2.1 Study site and survey locations

**FIGURE 1:**
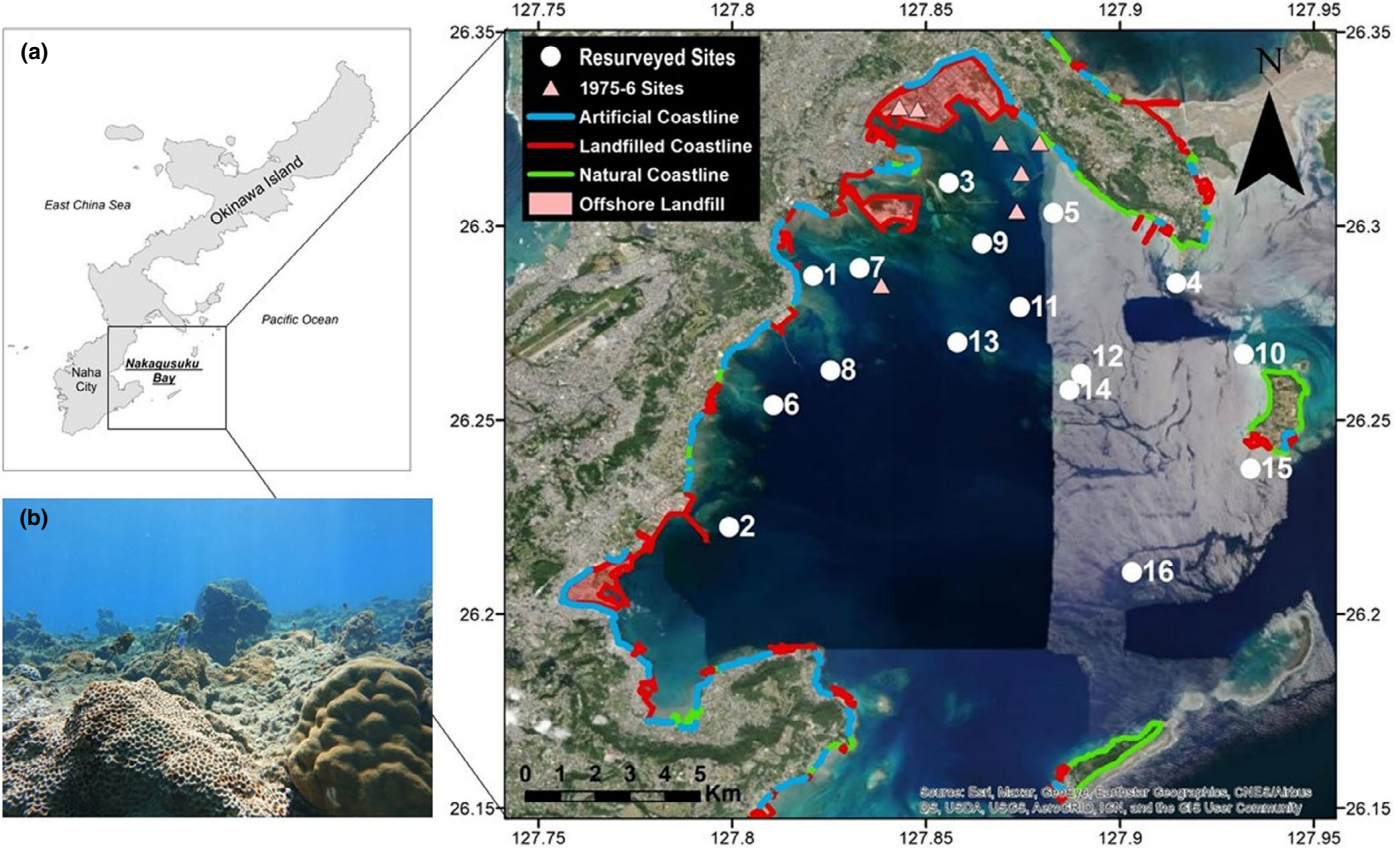
(a) Map of Nakagusuku Bay and its position on Okinawa Island. Sites that were surveyed in 1975–1976 and then resurveyed in 2018 are represented with a solid white circle and numbered according to distance from the coastline of the main island. Sites that were only surveyed in 1975 represented with a pink triangle. Colors represent coastline development as of 2018, much of which occurred after 1977 (Masucci & Reimer, 2019). (b) Photograph of a typical coral reef site (site 12) in 2018 showing a predominance of massive and encrusting corals

Surveys were conducted across Nakagusuku Bay, Okinawa, Japan (26.25°N 127.84°E) in 1975–1976 and repeated in 2018 (Figure 1). Nakagusuku Bay covers multiple marine habitats, including coral reefs, seaweed beds, and tidal mudflats. It forms a large part of an Ecologically or Biologically Significant Marine Area identified by the Ministry of Environment, and it is home to multiple endemic species (Japan Coral Reef Society, 2004). The bay has an average depth of 10–15 m, covers 220 km^2^, and faces east, and thus is impacted by yearly tropical typhoons (Rudolph et al., 1975). During World War Two, it was used as a major port, but significant development of the surrounding coastline did not occur until Okinawa was returned to Japan in 1972 (Kuwahara, 2012). The bay is now surrounded almost completely by urban areas and includes a large US naval base, a natural gas power plant, and multiple large areas of reclaimed land (Masucci & Reimer, 2019).

Initial surveys were conducted between 1975 and 1976 (Yamazato & Nishihara, 1977) on patch reefs in the northern half of Nakagusuku Bay. Yamazato and Nishihara (1977) reported an accurate topographical map including reef shape, depth contours and the relation of sites to the coastline and other sites that enabled us to replicate the surveys (Figure S1a,b). To resample the reef sites, we determined their coordinates by georeferencing the original maps with available satellite imagery. As each of the small patch reefs had a distinctive shape and was surrounded by areas of bare sand seafloor, they could be accurately located by boat sonar imagery when at the coordinate location. Three of the sites investigated in 1975–1976 were found to be landfilled in 2018 (Figure 1).

### 2.2 Coral surveys

Between December 1975 and April 1976, species and abundance of hermatypic corals present were recorded by visual observation during SCUBA dives at each major habitat (reef, reef slope, reef base/ bottom), of which depth was recorded. To calculate coral species percentage cover, 1 × 1 m quadrats were set at 1–3 points per site covering the major reef habitats and depth range. In the case of shallow reefs, the quadrat was set only on reefs, or only reefs and bottoms, and in the case of deeper reefs, the quadrats were also set at some reef slopes. The depth, coral species, number of colonies, and percentage coverage within the quadrat were recorded. Photographs of representative reefs at some of the sites were also captured (Figure S1).

The surveys were repeated between June and October 2018 at 16 of the remaining sites. These sites were selected as they were also the location of fish surveys in 1975–1976 and are still accessible by boat. Sites ranged from shallow near‐shore sites with a maximum depth of 1.8 m to offshore reef crest sites with a maximum depth of 36 m. Twenty 1 × 1 m quadrats were randomly placed across depths matching those of the original surveys for each site. A photograph was taken of the whole quadrat to estimate live coral cover, and then of each colony within the quadrat. Using these images, corals were identified to genus level using Indo Pacific ID guides, and coral cover was determined using CPCe software (Kohler & Gill, 2006).

### 2.3 Fish surveys

Fish surveys were also conducted between September 1975 and February 1976 at 31 sites, 23 of which were the same as the coral sites described above (Arasaki & Ida, 1977). A 50 m transect was extended from the shallowest point of the reef to the deepest point in a random direction. The width of the transect was not recorded. Fish species abundance observed while swimming along the transect was recorded, as well as the depths and the reef profiles of the surveys. Survey dives lasted 30–75 min, but it is unclear if the transect surveys lasted the whole length of the dive. Sub‐benthic and cryptic species were not recorded as their observability is low using these methods.

Between July and October 2018, replicate fish surveys were conducted alongside coral surveys. Fish surveys were not conducted at two of the sites where coral data were collected (sites two and six) due to weather‐related constraints. We recorded five 2 × 10 m video transects, at the maximum, middle, and minimum depths of those of the original surveys, resulting in 15 transects per site. Videos were recorded while swimming at a constant speed close to the edge of the reef slope, or the top of the reef, depending on topography of the site. After each 10 m transect, we then swam 10 m without recording to avoid double counts between transects. The individual (alive and dead) reef structures were often small, so the transect length was chosen to allow for replicates while avoiding surveying over bare sandy bottom. Fish were identified to species level from videos, and species present at each site were recorded.

### 2.4 Data analyses

All analyses were conducted using R (R Core Team, 2020), and all plots were constructed using the R package “ggplot2” (Wickham, 2011). Prior to any analyses the 1970s scleractinian coral genera and fish species were verified using online repositories, and species and genera were reassigned to their current correct names if needed. If a fish species had been split into multiple new species, these names were then checked in the FishBase online database (www.fishbase.org) and the species with the most appropriate geographical range was selected. Similarly, for taxonomic splitting of scleractinian coral genera, genera were checked against the Japanese Ministry of Environment coral surveys (Japan Coral Reef Society, 2004), and the most appropriate genus was selected according to range.

### 2.5 Coral coverage and depth change

Using the scleractinian coral quadrat data, we calculated total coral coverage per site (mean percentage coverage across the site‐specific quadrats), mean genus abundance per site, and relative abundance of each coral genera across both time points. The relationship between change in coral coverage and distance from Okinawa Island's coastline was explored using a linear model. Distance from coastline was taken to be a proxy from distance from urbanization and anthropogenic development, as the main Island's coastline has become largely non‐natural since 1977 (Masucci & Reimer, 2019). A two‐way ANOVA was performed to determine the effect of survey year and coral genera on the depth at which coral colonies were found. We also calculated the average genus depth value across all sites for both years, and coral genera were categorized into three depth categories: “shallow” <5 m, “medium” 5–8 m, and “deep” >8 m groups.

### 2.6 Coral genera and fish species community analyses

While the methods in 1975/1976 are well described, the exact sampling effort was not reported. Differences in sampling efforts could compound our results in such a way that we cannot be sure whether differences in trait communities are due to community transformation or survey methods. To compare sampling effort between time periods, we performed rarefaction analyses with extrapolation using the “iNEXT” R package (Hsieh et al., 2016). We plotted extrapolated species accumulation curves and sample coverage (sample completeness), based on 1975–1976 and 2018 incidence data across the whole study area, for coral genera and fish species. To explore accumulation patterns across sites, we also plotted site‐based accumulation curves for coral genera and fish species richness for 1975–1976 and 2018 using the “specum” function from the R “Vegan” package (Oksanen et al., 2019). Sites were added in a random order over 100 permutation, and genus and species richness was calculated per site for scleractinian coral and fish, respectively, for both time periods (1975–1976 and 2018).

We conducted multiple statistical analyses to assess if the communities had changed between the years. All community analyses were conducted using the R package “vegan” (Oksanen et al., 2019). We applied a paired *t*‐test to site‐based richness values for both coral and fish to test if the difference between years was statistically significant. To visualize the changes in fish and coral communities at each site between years, we conducted a Principal Component Analyses (PCA). Presence and absence data were used for both corals and fish in order to compare between taxa. We then ran a PERMANOVA on the Bray‐Curtis dissimilarity matrices using the “adonis2” function to test for significant differences between communities and years. Finally, we used the “simper” function to run a post‐hoc test to explore which species/genera were driving these differences.

### 2.7 Fish community generalization

To explore if fish communities became more generalized in 2018 compared to 1975–1976, we used a species generalization index (SGI) calculated from a dataset of global fish surveys in relation to benthic habitat classes (for detailed methods see Stuart‐Smith et al., 2021). SGI data was available for 242 species out of a total of 306 species observed in our surveys. Species that did not have SGI data were excluded for this part of the analysis. The SGIs are an indicator of fish habitat niche, with larger values corresponding to a larger niche and thus a more generalist species (Stuart‐Smith et al., 2021). The community generalization index (CGI) of each site was calculated using the mean SGIs of fish species present for both years (Stuart‐Smith et al., 2021). To see if there was a significant increase in CGI between the years, we analyzed the CGIs of the sites using a paired sample t‐test.

### 2.8 Functional trait‐based community space

We created trait databases for all our surveyed fish species and coral genera to understand how the Nakagusuku Bay communities had changed functionally. An array of morphological, behavioral, and phenological traits were selected to represent functional niche, and thus roles within the ecosystem. For fish, we selected the following traits: maximum length, depth range, trophic level, behavioral aggregation, water column position, spawning mode, and parental mode. These traits infer what the species can eat, where they can survive, and how specialized their ecology is, which can be critical parameters when identifying drivers of community change (Mouillot et al., 2014; Nock et al., 2016). Traits were collated from online databases including FishBase as well as from extensive literature searches for local endemic species. For scleractinian corals, traits were downloaded at the species level from the Coral Traits database (https://coraltraits.org/) (Madin et al., 2016) for all species present in Japan as based on Ministry of Environment surveys (Japan Coral Reef Society, 2004). The mean of the continuous numeric traits was calculated for each genus, and for categorical traits, the value that occurred the most was selected. We used the traits coloniality, maximum corallite width, typical growth form, water clarity preference, wave exposure preference, sexual system, larval development, growth rate, oocyte size, and depth range.

The function “gowdis” from the “FD” package was used to compute the Gower dissimilarity matrix from the species/genera by trait matrices (Laliberté et al., 2014). We used the Gower dissimilarity index because our trait data contained a mix of categorical and continuous traits and contained missing values for rarer species and genera. We then ran a Principal Coordinate Analysis (PCoA) on the distance matrices using a Cailliez correction to visualize traits in multivariate space. By plotting the PCoA values of the overall community, and then the 1975–1976 and 2018 communities separately, we aimed to identify if there had been a shift in trait space. We conducted this analysis comparing both the species/genera present across the whole bay between the years, and for individual sites between the years. We then calculated individual hull areas for each site for both time periods using the function “areapl” from the “splancs” package (Bivand et al., 2017) and analyzed the change in area between years using a paired sample t‐test.

## 3 RESULTS

### 3.1 Coral cover community change

**FIGURE 2:**
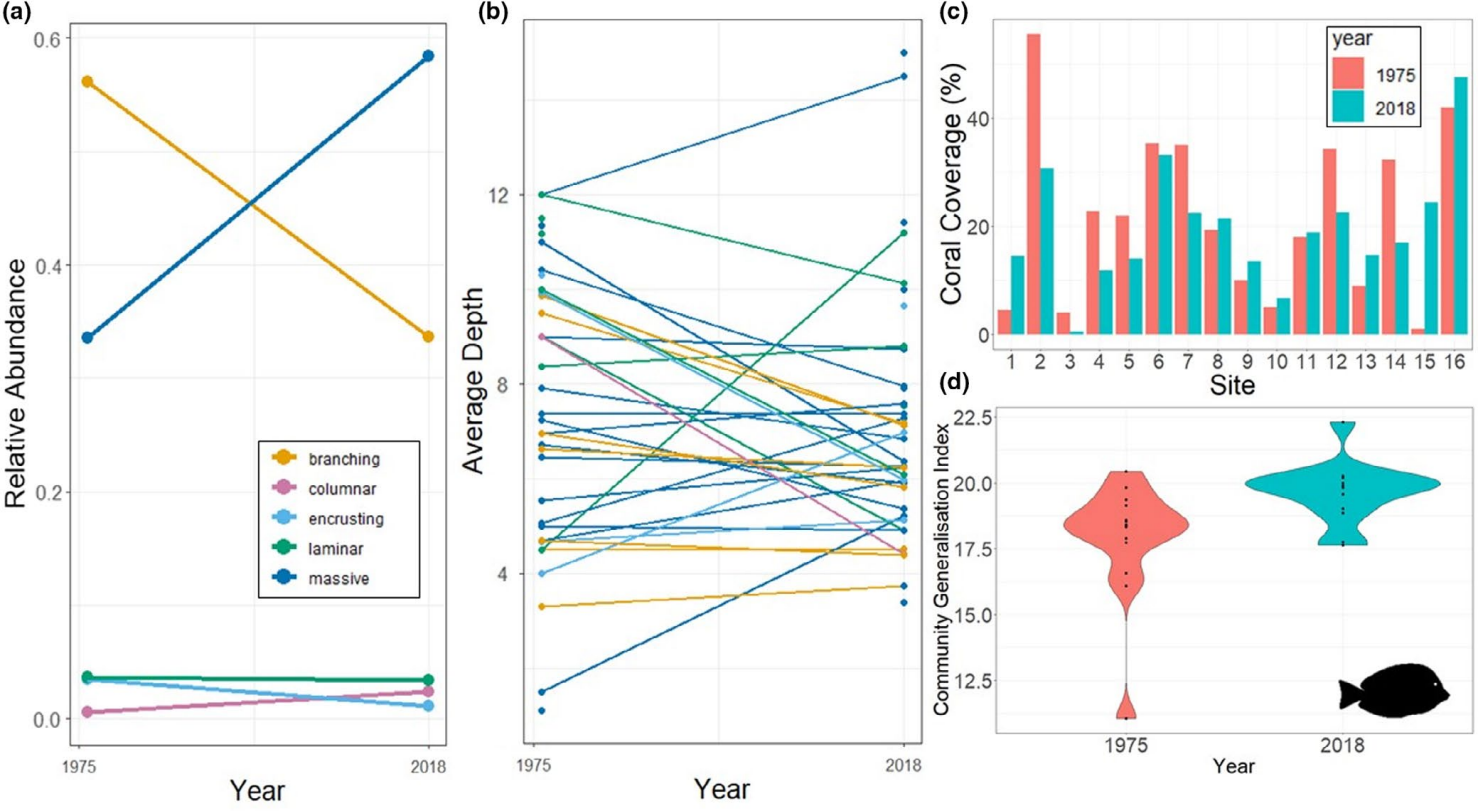
Summary of community reassembly in Nakagusuku Bay, showing (a) change in coral relative abundances between the years 1975–1976 and 2018 calculated across all sites for the main coral growth forms, (b) changes in the average depth at which each coral genus was found in the years 1975–1976 and 2018. Each line represents a coral genus, colored by its most common growth form. (c) Changes in average percentage coral coverage at each of the sites in the years 1975–1976 and 2018. (d) Change in the fish community generalization index between the years 1975–1976 and 2018 across all sites

Live coral percentage cover in 1975–1976 ranged from a minimum of 1% at site 15 to a maximum of 56% at site 2, and in 2018 ranged from a minimum of <1% at site 3 to a maximum of 48% at site 16. Eight of the sites (site 2, 3, 4, 5, 6, 7, 12, 14) experienced a decline in coral coverage, with the remaining eight sites (1, 8, 9, 10, 11, 13, 15, 16) experiencing an increase (Figure 2c). In general, as the site's distance from Okinawa Island increased, so did the change in coral coverage (*R* ^2^ = 0.13, *F*(1,14) = 3.29, *p *= .09) (Figure S2). The exceptions to this pattern were site one, which increased in coverage but was closest to the coast, and sites 12 and 14 which had much higher losses in coverage.

**FIGURE 3:**
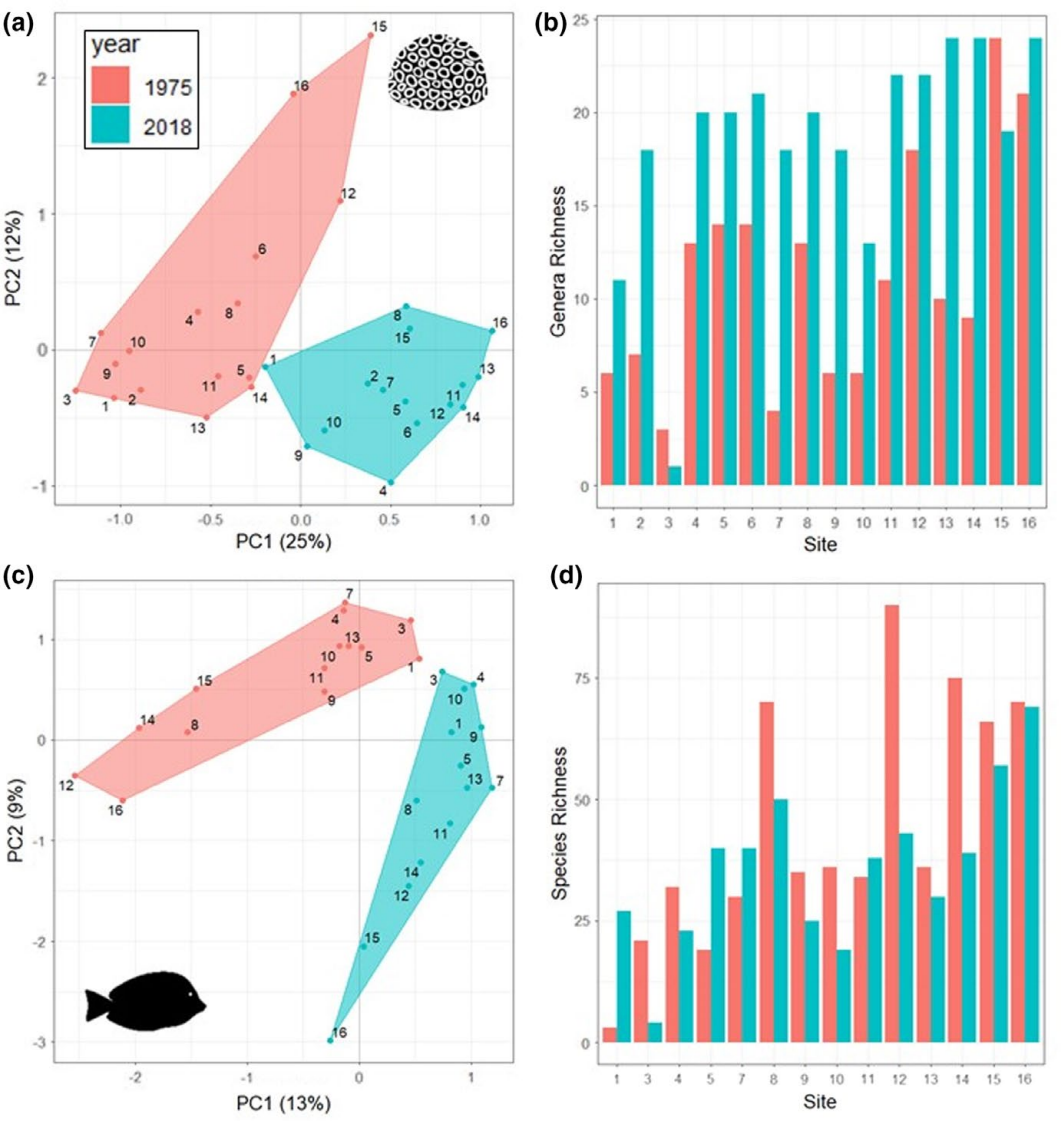
Summary of species and genera community changes for fish and coral across individual sites in Nakagusuku Bay, Okinawa. Sites are numbered according to distance from the coastline of the main island. (a) Principal component analyses of coral genera present at each site for the years 1975–1976 and 2018. (b) Number of coral genera present at each site for the years 1975–1976 and 2018. (c) Principal component analyses of fish species present at each site for the years 1975–1976 and 2018. (d) Fish species richness at each site for the years 1975–1976 and 2018

Although coral genera richness appeared to increase or be maintained across all the sites except site 15 (Figure 3b), there was a shift in dominance from coral genera with branching growth forms to ones with massive growth forms (Figure 2a). *Acropora* corals accounted for 25% of the corals surveyed in 1975–1976 but dropped to 4% in 2018. *Porites* corals accounted for 20% of the corals in 1975–1976 and increased to 24% in 2018. The top five genera with the largest increases in relative abundance all had massive growth forms: *Dipsastraea* (+10%), *Cyphastrea* (+7%), *Astreopora* (+6%), *Favites* (+5%), and *Porites* (+4%). Four out five of the genera with the largest decreases in relative abundance had branching growth forms: *Acropora* (−21%), *Stylophora* (−3%), *Seriatopora* (−2%), and *Pectinia* (−1%).

### 3.2 Coral depth distribution analyses

The average depths at which each coral genera was found differed significantly (ANOVA: *F* = 12.835, *df* = 45,4218, *p *< .001). Overall, the change in depth between the years 1975–1976 was found to be insignificant (ANOVA: *F* = 0.830, *df* = 1,4218, *p *= .326), but the interaction term between year and genera was found to be significant (ANOVA: *F* = 4.116, *df* = 30, 4218, *p *< .001). The overall pattern suggests that coral genera that were once more abundant at shallower depths <5 m shifted deeper, and genera that were more abundant at deeper depths >8 m shifted shallower (Figure 2b). Corals that had a medium average depth between 5 and 8 m in the 1970s have largely remained at similar depths in the 2018 survey. When categorizing genera by their average depths (shallow =< 5 m, medium = 5–8 m and deep>8 m) in 1975–1976, nine genera were found to be shallow, 11 medium, and 18 deep. In 2018 this distribution shifted to eight shallow, 22 medium, and nine deep genera.

### 3.3 Coral genera and fish species community analyses

Results from the extrapolated rarefaction analyses showed that sample coverage estimates were similar for both years, with coral estimates being 97.6% and 99.7% and fish estimates being 89.8% and 92.1% for 1975–1976 and 2018, respectively (Figure S3). This result supports the notion that we likely succeeded in replicating sampling strategies and effort adequately, generating a comparable sample. Thus, differences in community composition are highly likely to be attributable to community change rather than sampling protocol.

The richness of coral genera across all sites significantly increased between 1975–1976 and 2018 (*t* = −5.83, *df* = 14, *p *< .01), with an increase at 14 of the 15 sites (Figure 3b). However, across the whole bay, total coral genera richness only increased from 38 to 40, and when taking into account the slightly different sampling efforts, there was no overall change in richness (Figure S3). In contrast, fish species richness decreased at 10 of the 14 of the sites, but overall changes between years were non‐significant (*t* = 1.54, *df* = 13, *p *= .15) (Figure 3d). Between the years, the total number of fish species remained stable at 198, and extrapolations of richness to full sample coverage confirmed that there was no significant difference in richness between years (Figure S3). Site‐based accumulation curves for fish genera and coral species were stable in both periods and showed similar slopes, although the curves from 1975 to 1976 had larger confidence intervals, suggesting that in the past, richness was more variable across sites (Figure S4). For both fish and corals, the patterns in richness between sites remained similar across the years (Figure 3). The sites with a higher richness in the 1970s generally still had a higher richness in 2018. Both the fish and coral PCAs revealed that community composition was distinctly different between 1975–1976 and 2018, with two distinct clusters (Figure 3a,c). The sites clustered similarly for the 1975–1976 coral and fish cluster, and the 2018 fish clusters, with sites 12, 15, and 16 seeming to have more unique compositions. The 2018 sites for coral were more closely clustered together, suggesting potential homogenization of coral communities.

Coral communities differed significantly between the years at each site (PERMANOVA: *F* = 7.94, *R* ^2^ = .21, *p *< .01) (Figure 3a, Table S1.). The SIMPER analyses did not identify any genera that significantly drove these changes. *Turbinara* accounted for the highest percentage of dissimilarity at 5%, followed by *Astreopora* (4.9%), *Psammocora* (4.4%), *Astrea* (4.3%), and *Pavona* (4.1%) (see Table S3 for full list).

Fish communities also differed significantly between years (PERMANOVA: *F* = 5.53, *R* ^2^ = .17, *p *< .01) (Figure 3c, Table S2). The results from the SIMPER analyses showed that there were no characteristic species or groups that were driving these changes. For example, *Acanthurus nigrofuscus* accounted for the highest percentage of dissimilarity between the years at 1.3% followed by *Ctenochaetus binotatus* (1.1%), *Sargocentron rubrum* (1.1%), *Meiacanthus* sp. (1.1%), *Chaetodon plebeius* (1.1%), and *Siganus virgatus* (1%) (see Table S4 for full list). However, considering there were a total of 309 species surveyed overall, and 65 of these species accounted for 50% of the variation between the years, there were still disproportional effects.

Overall, there was a significant increase in the community generalization index (CGI) between the 1975–1976 and 2018 (*t* = −2.72, *df* = 13, *p *= .02). The fish community transitioned to contain more habitat generalists at 10 of 14 sites (Figure 2d), and the remaining sites only had a small decrease in CGI (i.e., signs of a shift in the community consistent with specialization). There appeared to be no spatial patterns in CGI change.

### 3.4 Functional trait‐based community space

**FIGURE 4:**
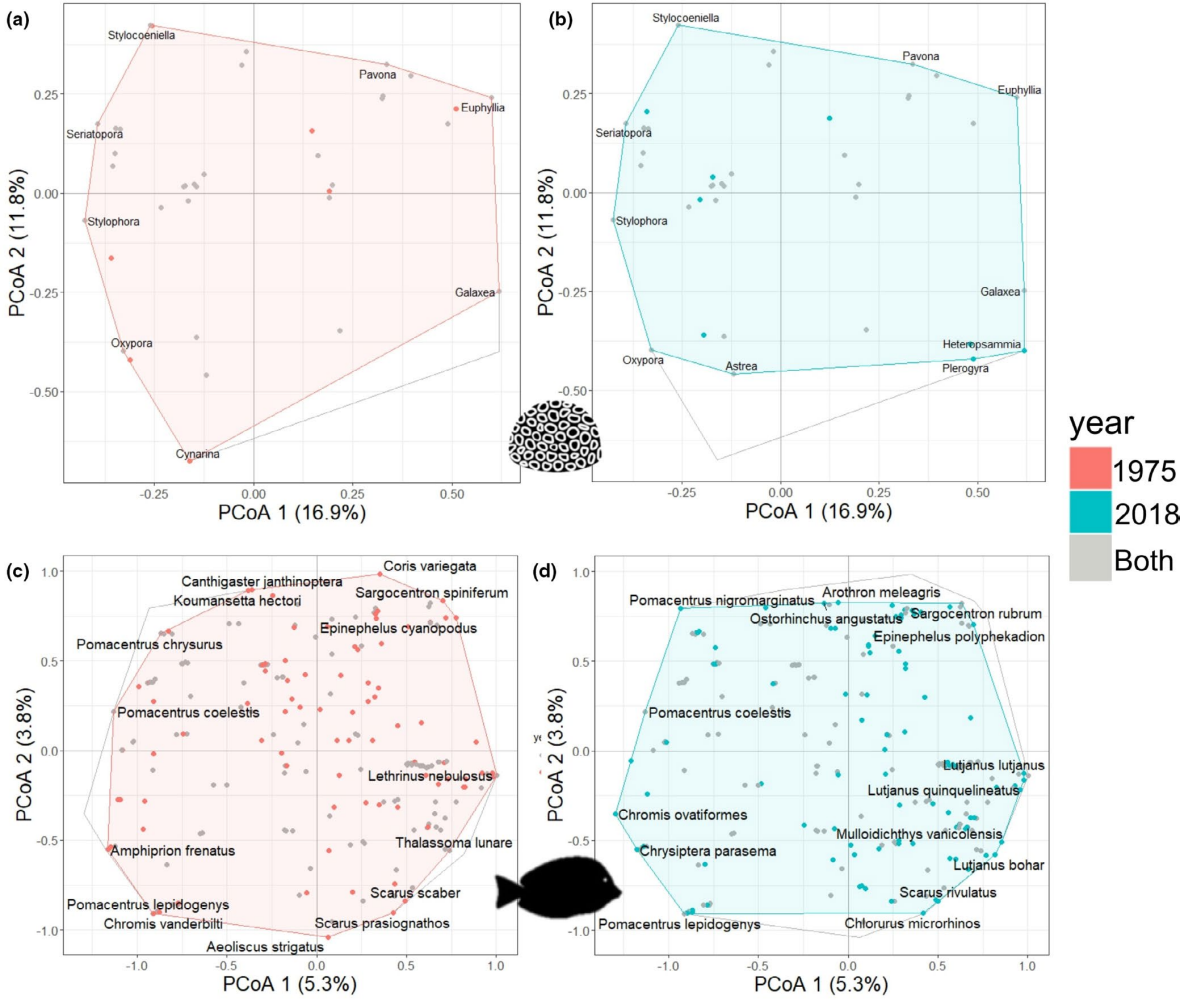
Summary of changes in functional trait space in Nakagusuku Bay, Okinawa, between 1975 and 2018. (a) Gower distance‐based principal coordinate analyses (PCoA) of coral traits present across the whole study area for 1975–1976. (b) PCoA based on the Gower distances of coral traits for 2018. (c) PCoA based on the Gower distances of fish traits for 1975–1976. Points represent individual fish species. (d) PCoA based on the Gower distances of fish traits for 2018. Gray convex hull represents overall site wide trait space across both years, colored hull represents year specific trait space. Colored points represent species or genera present only in the corresponding year, whereas gray points represent species or genera present across both study years

When using PcoA to visualize the changes in the overall bay‐wide coral community trait structure over time, the first two PcoA axes cumulatively explained 28.6% of the overall inertia (Figure 4a,b). The trait space was slightly altered by the loss of the genera *Mycedium* and *Cynaria* in 2018, and the addition of the genera *Trachyphyllia*, *Heteropsammia*, and *Plerogyra*. Between the surveys, genera turnover occurred evenly across the trait space. The hull area of the coral trait space of individual sites increased slightly overall (*t* = −2.6, *df* = 15, *p *= .02) with only two sites (1 and 10) showing a shift in space (Figure S5a,b). Similarly, when using PcoA to visualize how the overall fish community trait structure has changed over time, the first two PcoA axes cumulatively explained 9.1% of the projected inertia (PcoA 1 = 5.3% and PcoA 2 = 3.8%) (Figure 4c,d). Between 1975 and 2018 there was very little change in trait space, and species turnover seems to be spread evenly across the space. When looking at the individual sites, site one experienced a large increase in trait space, and site three experienced a large decrease (Figure S6a,b). However, the rest of the sites stayed largely the same, the overall change in trait space hull area between the years was not significant (*t* = 0.39, *df* = 13, *p *= .70). Species that were present in 1975–1976 and then lost in 2018 that contributed to a contraction of trait space included *Epinephelus quoyanus*, *Epinephelus cyanopodus*, *Epinphelus fasciatus*, *Canthigaster janthinoptera*, *Koumansetta hectori*, and *Aeoliscus strigatus*. Species that were gained in 2018 compared to 1975–1976 that expanded the trait space included *Chromis alleni*, *Chromis ovatiformes*, *Pomacentrus nigromarginatus*, *Lutjanus gibbus*, *Lutjanus bohar*, and *Gnathodentex aureolineatus*.

## 4 DISCUSSION

Over the past four decades, the coral reef communities of Nakagusuku Bay have been subject to the effects of extensive anthropogenic disturbances, most noticeably coastal development and three global bleaching events. Thus, we expected a loss of coral cover, a reduction in genera and species richness, and a community shift towards more stress‐tolerant species that would be associated with a loss of ecosystem functions. By contrast, we discovered that while a significant change in fish and coral community compositions occurred over time, the range of functions remained stable. The overall community shift across the bay was characterized by a turnover of species and genera across the entire functional trait space, not a shift to groups with similar traits and associated contraction of trait space. However, we found that the fish communities have become dominated by habitat generalists, indicating a homogenization of the habitat association trait, which was not considered in our trait‐space analysis. We observed uneven declines in coral coverage, with associated inconsistent patterns in richness changes of both fish and coral. Our results also indicate “depth compression’ in corals, and a shift in dominance from branching to massive/submassive coral morphologies. Our results support the hypothesis that the combined anthropogenic stressors of urban coral reefs alter community structure towards more generalist species, with specific findings of local depth shifts. But, thus far, these stressors do not appear to have resulted in a loss of ecosystem function.

Coral coverage declined at half of the sites. These sites were predominantly located near to landfilled areas and non‐natural sections of the bay's coastline. This geographic pattern suggests that coral coverage in the bay may be highly susceptible to such localized anthropogenic impacts. In contrast, all but one of the sites experienced a uniform increase in coral genera richness, although bay wide richness remained stable. This increase could be explained by the observed shift in dominance from genera with branching to massive growth forms. Die offs of branching corals have been recorded globally as a result of bleaching events, and thus the loss of branching corals at our sites due to thermal stress could have allowed for the recruitment of a more diverse array of less competitive, yet more disturbance resilient genera which already persisted within the bay but in lower abundances (Adjeroud et al., 2018). However, although sampling effort was predicted to be comparable between the years across the whole bay, a level of uncertainty remains because detailed sampling methods were not reported in 1975, and despite our best efforts to take this into account with rarefaction analyses, we may have sampled differently in 2018.

Sites that increased in coral cover and richness did not increase in fish richness. Site specific reductions in fish species richness could be linked to the shifts in coral morphologies (Darling et al., 2017). Branching corals are more structurally complex than massive corals, so their loss at our sites may have reduced habitat availability for more specialized fish species (Richardson et al., 2018), as indicated by a significant increase across sites in the community generalization index. The sites also experienced significant shifts in fish species composition. Generalist species such as *Acanthurus nigrofuscus*, *Ctenochaetus binotatus*, and *Siganus virgatus* had very high SGIs, and the SIMPER analyses indicated that an increase in their presence across sites from 1975–1976 to 2018 (Stuart‐Smith et al., 2021).

Significant shifts in coral community composition between the years appear to be driven by the introduction of genera with species know to have tolerance to turbidity and bleaching such as *Turbinaria*, *Astreopora*, and *Psammocora* (Marshall & Baird, 2000). Although these genera were present at some sites in the 1970s, they were recorded across more sites in 2018. It is not possible to quantify with our data whether bleaching events or local disturbances are driving such shifts, as that would require time series data for ecological and environmental variables. However, our results match patterns observed on Singapore's urban turbid reefs over a 27 year continuous time series analysis, which attributed community change to both thermal stress events and turbidity (Guest et al., 2016). These reefs also experienced community turnover, although there was no evidence of shifts from branching to more stress tolerant species, possibly due to these shifts occurring prior to the beginning of monitoring. Most noticeably, Singapore's reefs underwent a “reef compression” (Guest et al., 2016), and we mirror this finding in Nakagusuku bay, with coral genera that were found deeper on average in the 1970s shifting shallower in 2018, and those found at shallower depths in 1975–1976 shifting deeper. This phenomena could be attributed to the effects of thermal stress events and bleaching, which are more pronounced in the shallows, resulting in higher mortality rates in the shallows (Chow et al., 2019; Guest et al., 2016). Deeper colonies may be thermally protected from bleaching, yet increased turbidity levels due to local stressors may reduce light levels past the critical point for phototrophic organisms (Browne, 2012). Our corroborative finding of “reef compression” suggests these types of community shifts could be typical for coastal coral reefs exposed to urbanization (Guest et al., 2016). With continuing coastal urbanization and thermal stress events, reef compression could continue to reduce suitable habitat area for coral reef species. If such compression occurs at a wider scale, it could result in significant losses of coral reef ecosystems and the species which depend on them.

Although community composition changed, the overall trait space for both fish and coral remained similar between the years. There was a turnover of species and genera, but these were lost and gained evenly across the trait space. Corals had high sample coverage between the years, with the recorded total richness being similar to the predicted total richness with extrapolated rarefaction curves. This pattern suggests that the observed turnovers captured the actual change in the community over this time period. However, fish had lower sample coverage between the years. When extrapolated to full coverage for both years, species richness increased. Although the total richness was not significantly different between years, these additional species may have increased the fish functional trait spaces. However, our current results indicate that for both fish and coral, species and genera that were lost were replaced with species and genera with similar traits. The turnover of taxa with similar traits suggests that their functional roles remained (McWilliam et al., 2020), inferring the maintenance of ecosystem functioning across the whole study area between the years (Mouillot et al., 2013). However, the trait‐based analyses also did not account for abundance. This could be important as, for example, *Acropora* was once the dominant coral genera but has now been reduced to just 4% of the total coral colonies surveyed. Although there are some remnant colonies, they will not be providing as much of the important functions as they once did (McWilliam et al., 2020). Yet, in terms of reef resilience, these remnant populations may be critical for the recovery of reefs post‐disturbance for both coral and fish (Kayal et al., 2018).

Trait space constancy could indicate that at the time of the original survey in 1975–1976 the study area was already subject to human disturbances. Corals and fish with unique traits that could only survive in “pristine” environments may have already been lost by the mid‐1970s due to the impacts of World War Two, leaving a suite of more generalist species (Omori, 2011). Under further degradation, we may not have observed further shrinkage, as if the initial trait‐based shift had already occurred, this new community in 1975–1976 may have been more resilient to further disturbances. Alternatively, for fish, trait space could have been maintained due to a shift from clear water reef specialists to sandy or mud bottom turbid specialists, especially in sites with a high loss of coral coverage (Brandl et al., 2016). This implies that functional losses are not linearly linked to disturbance gradients.

It must be noted that due to the historical nature of the 1975–1976 dataset, we updated survey methods in 2018 to increase robustness and statistical power for potential future surveys. Details of the original survey sites were meticulously recorded (Yamazato & Nishihara, 1977), allowing us to resample the exact locations. However, historical coral surveys were not carried out along transects, but with visual observation survey dives across representative reef habitats (reef, reef slope, and reef base). In 2018, we used quadrat methodology, standardizing our survey area and allowing us to calculate percentage cover. In 1975–1976, a small number of quantitative quadrat surveys were conducted with these same methods with much fewer replicates. Historical fish surveys were carried out across a transect length of 50m, matching our individual transect lengths in 2018. However, observability would have been influenced by transect width, swim speed, and visibility, details of which were not recorded. As the sites were all based on small patch reefs, it is likely that the original surveys covered a relatively large area in comparison to overall reef size, capturing an accurate representation of the communities. To ensure the exact reefs were included, and to increase the future repeatability of the surveys, we used standardized sampling procedures and increased replicate numbers of surveys across the same reefs. Both historical and current surveys sampled reef communities on singular dives lasting about 60 minutes, suggesting similar sampling efforts, and this was verified by the high similar sample coverage predictions in the rarefaction analysis (Figure S3). Abundance results would be more sensitive to sampling effort, but we only compared presence/absence of taxa, except for coral percentage cover where the quadrat method was directly comparable. Presence/absence data is well used in community ecology, especially in temporal studies, as they show species losses and gains, providing insight into changing ecological processes (Legendre, 2019).

Temporal comparisons are difficult where the historical sampling process was not described in enough detail, as differences may be attributable to methods rather than community change. We accounted for this to the best of our ability by performing statistical tests to show sampling effort, supporting the validity of our approach. Furthermore, our results show that many species were not observed in 2018, despite predicted equal or higher sampling efforts (Table S1, Table S2). If our findings were due to differences in methods, we would expect to find the same species that were recorded in the 1970s, plus additional species with the increased sampling effort. Contrastingly, we found that seven coral genera and 107 fish species were not recorded in 2018, suggesting they truly disappeared from our sites. The loss of a large proportion of taxa strengthens the case for disturbance‐induced community turnover and reduces the likelihood that the results were due to altered sampling protocols. However, working with historical data holds challenges related to how scientific methods and technology have changed over time. Given the differences in our methods, we acknowledge that species richness differences between historical and modern surveys may be due to challenges with comparing fish data from different surveys, relating to observer errors, potential differences in sampling efforts, or site differences, despite our best efforts to minimize and avoid such issues. Comparability issues such as these are a common when using older survey data collected before the introduction of standardized sampling methods (Tingley & Beissinger, 2009). However, such data should not be discounted for science, as it provides an invaluable resource for understanding community change and shifting historical baselines (Richards et al., 2008).

Our research highlights that urban turbid reefs such as those in Nakagusuku Bay may have underlying resilience to disturbances, as we did not observe large losses in fish and coral richness, and coral coverage was maintained across most sites. This could be a relic due to extinction lag, with low abundances of species that survived through disturbances persisting in unfavorable conditions with reduced growth, survival and reproduction (Graham et al., 2007). Shifts to communities dominated by massive and sub‐massive corals could be an early indicator of a tipping point to an alternate stable state (Mellin et al., 2019). Corals with this growth form are still vulnerable to longer term, severe thermal stress events, which are predicted to recur at an increasing frequency in the near future. If these corals experience high levels of mortality, they are unlikely to be able to recover due to their slow growth rates and reproductive strategies (Darling et al., 2012). However, reefs may be able to survive at mid‐depths, maintaining ecosystem functions and providing remnant populations for post‐disturbance recovery. Disturbed reefs may have reduced structural complexity, but our results indicate that the corals and associated fish species are continuing to provide critical functional roles. Thus, under urbanization and thermal stress, coral reef communities are likely to be significantly altered but not disappear completely (Robinson et al., 2019). The shifts towards urban turbid reefs is becoming increasingly common worldwide (Heery et al., 2018), and these functioning reef systems may have overlooked or underappreciated conservation value.

## CONFLICT OF INTEREST

All authors declare that they have no conflicts of interest.

## AUTHOR CONTRIBUTIONS

**Katie M. Cook:** Conceptualization (equal); Data curation (equal); Formal analysis (lead); Funding acquisition (supporting); Investigation (equal); Methodology (equal); Software (lead); Visualization (lead); Writing – original draft (lead); Writing – review & editing (equal). **Hirotaka Yamagiwa:** Conceptualization (equal); Data curation (equal); Formal analysis (supporting); Funding acquisition (supporting); Investigation (equal); Methodology (equal); Project administration (lead). **Maria Beger:** Conceptualization (equal); Funding acquisition (equal); Investigation (supporting); Project administration (supporting); Supervision (equal); Validation (equal); Writing – review & editing (lead). **Giovanni Diego Masucci:** Investigation (supporting); Resources (supporting); Writing – review & editing (supporting). **Stuart Ross:** Data curation (supporting); Formal analysis (supporting). **Hui Yian Theodora Lee:** Conceptualization (supporting); Formal analysis (supporting); Investigation (supporting); Methodology (supporting). **Rick D. Stuart‐Smith:** Resources (supporting); Validation (supporting); Writing – review & editing (supporting). **James Davis Reimer:** Conceptualization (equal); Funding acquisition (equal); Investigation (equal); Methodology (supporting); Project administration (equal); Resources (lead); Supervision (equal); Validation (equal); Writing – review & editing (equal).

## Supporting information

Supplementary Material

## Data Availability

The data that support the findings of this study are openly available in the Dryad Digital Repository at https://doi.org/10.5061/dryad.9ghx3ffk7. Fish SGI values are accessible through the RLS Reef Species of the World online species database (https://reeflifesurvey.com/species/search.php).
