# Supplementary material for: A community and functional comparison of coral and reef fish assemblages between four decades of coastal urbanisation and thermal stress
Source: Ecol Evol. 2022 Mar 22;12(3):e8736. doi: 10.1002/ece3.8736 (PMC8939291; doi:10.1002/ece3.8736)
Supplement: Supplementary file 1 — Supplementary Material [file ECE3-12-e8736-s001.docx]

**Supplementary Materials- Coral and reef fish community and functional change over 43 years of coastal urbanisation and thermal stress.**

Authors: Katie M Cook , Hirotaka Yamagiwa, Maria Beger, Giovanni Diego Masucci, Stuart Ross, Lee Hui Yian Theodora, Rick D. Stuart-Smith & James Davis Reimer.

**
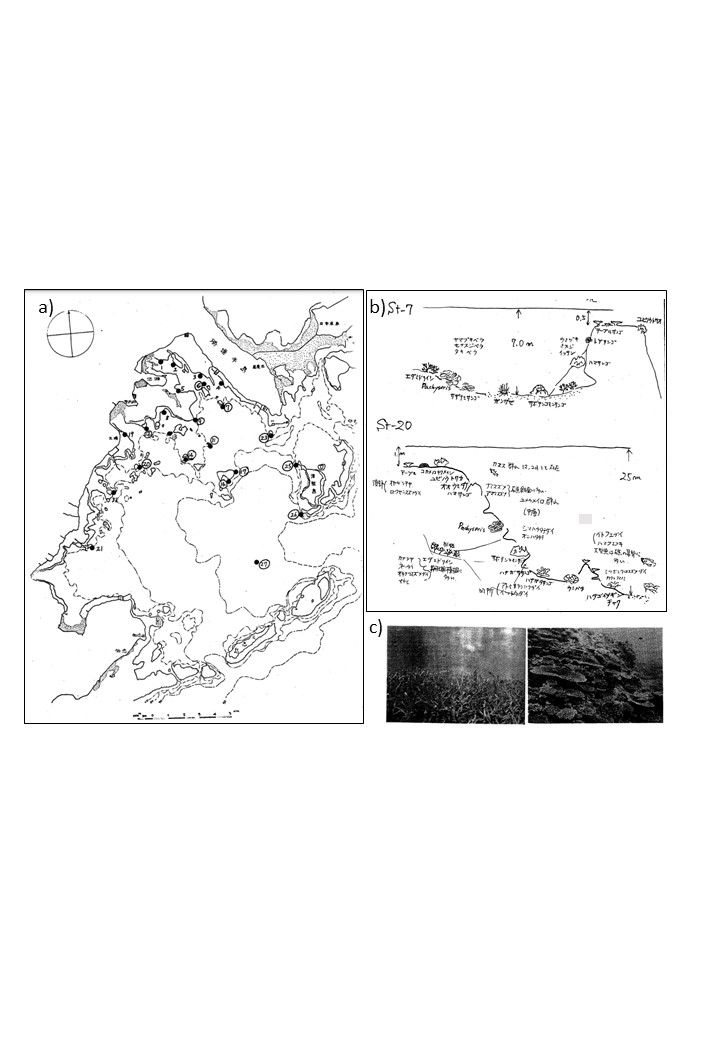
**

**Supplementary Fig. 1)** **a)** Original survey site map from Yamazato and Nishihara (1977). This map was georeferenced and used to determine GPS co-ordinates of each site for replicate surveys in 2018. Sites names were changed in 2018 to reflect distance from the Okinawa main island. **b)** Diagrams of Site 7 (now Site 5) and Site 20 (now Site 8) showing distinctive topography, depth and representative coral genera present in 1977 (Yamazato and Nishihara 1977). Such diagrams were available for each of the sites that resurveyed, enabling accurate relocation of survey sites. **c)** Images taken of coral assemblages in Nakagusuku Bay during 1975-6 (Yamazato and Nishihara,1977). Images show dominance of plating and branching corals, which have significantly reduced in coverage in the present day.

**
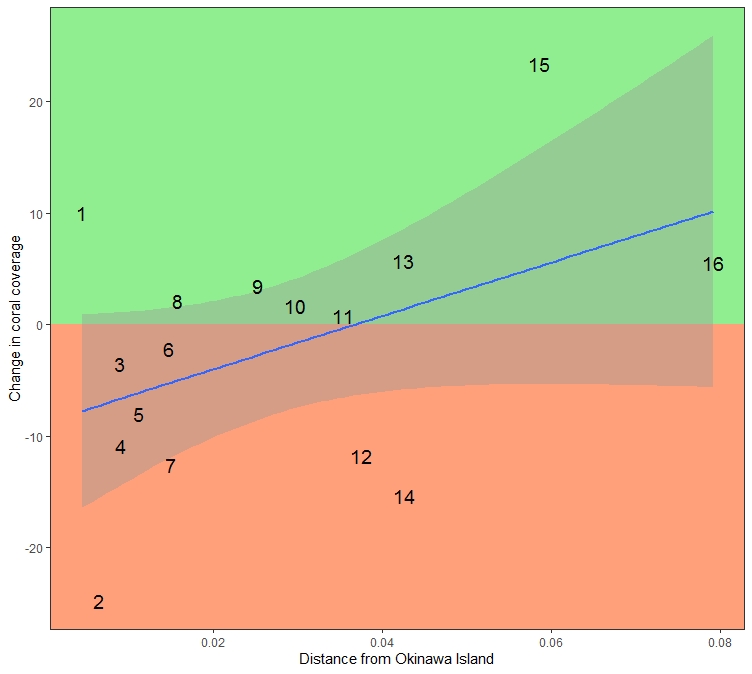
**

**Supplementary Fig. 2)** Relationship between site distance from Okinawa main island coastline and change in coral coverage between 1975-6 and 2018. Line shows linear model with 95% confidence intervals (R^2^=0.13, F(1, 14)= 3.29, P=0.09). Numbers indicate site names.


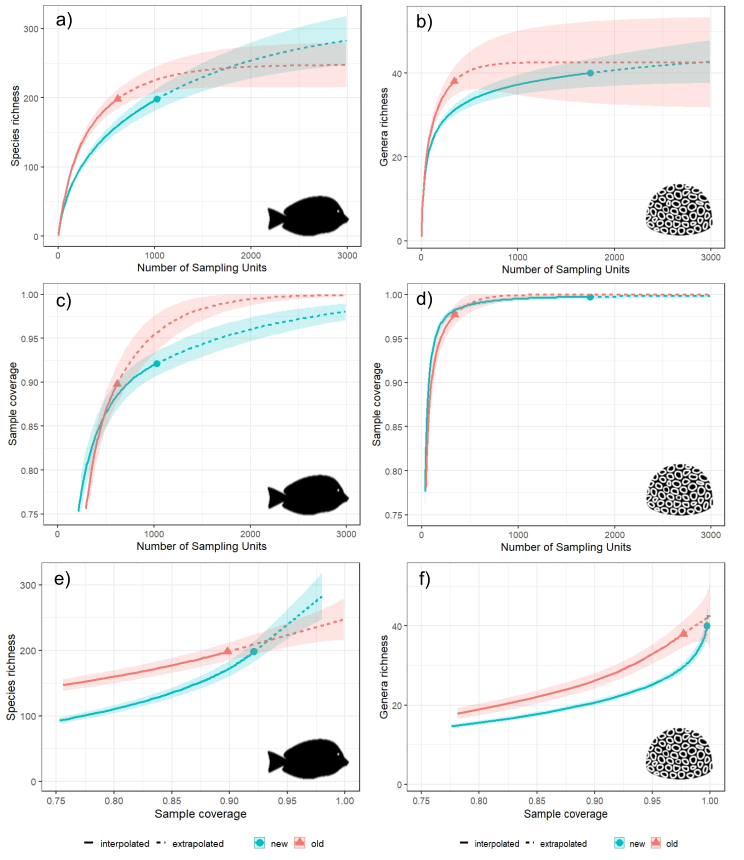


**Supplementary Fig. 3)** Rarefaction (solid line segment) and extrapolation (dotted line segments) sampling curves with 95% confidence intervals (shaded areas) for the fish species and coral genera. **a, b)** Sample size – based curves for fish species and coral genera, **c, d)** Sample completeness curves based on fish species and coral genera, **e, f )** Coverage-based curves for fish species and coral genera.

**
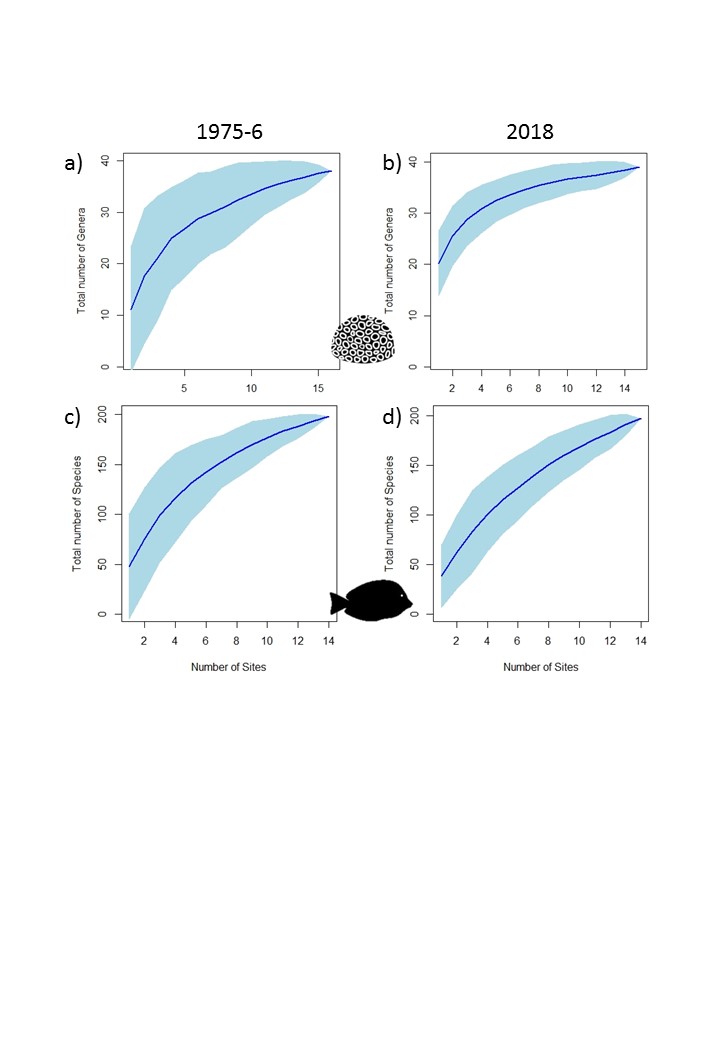
**

**Supplementary Fig. 4)** Genera and species richness accumulation curves produced by sequentially adding sites in a random order and calculating total richness, run over 100 permutations. Light blue bands represent 95% confidence intervals. **a)** Coral genera richness accumulation curve for 1975-6. **b)** Coral genera richness accumulation curve for 2018. **c)** Fish species richness accumulation curve for 1975-6. **d)** Fish species richness accumulation curve for 2018.


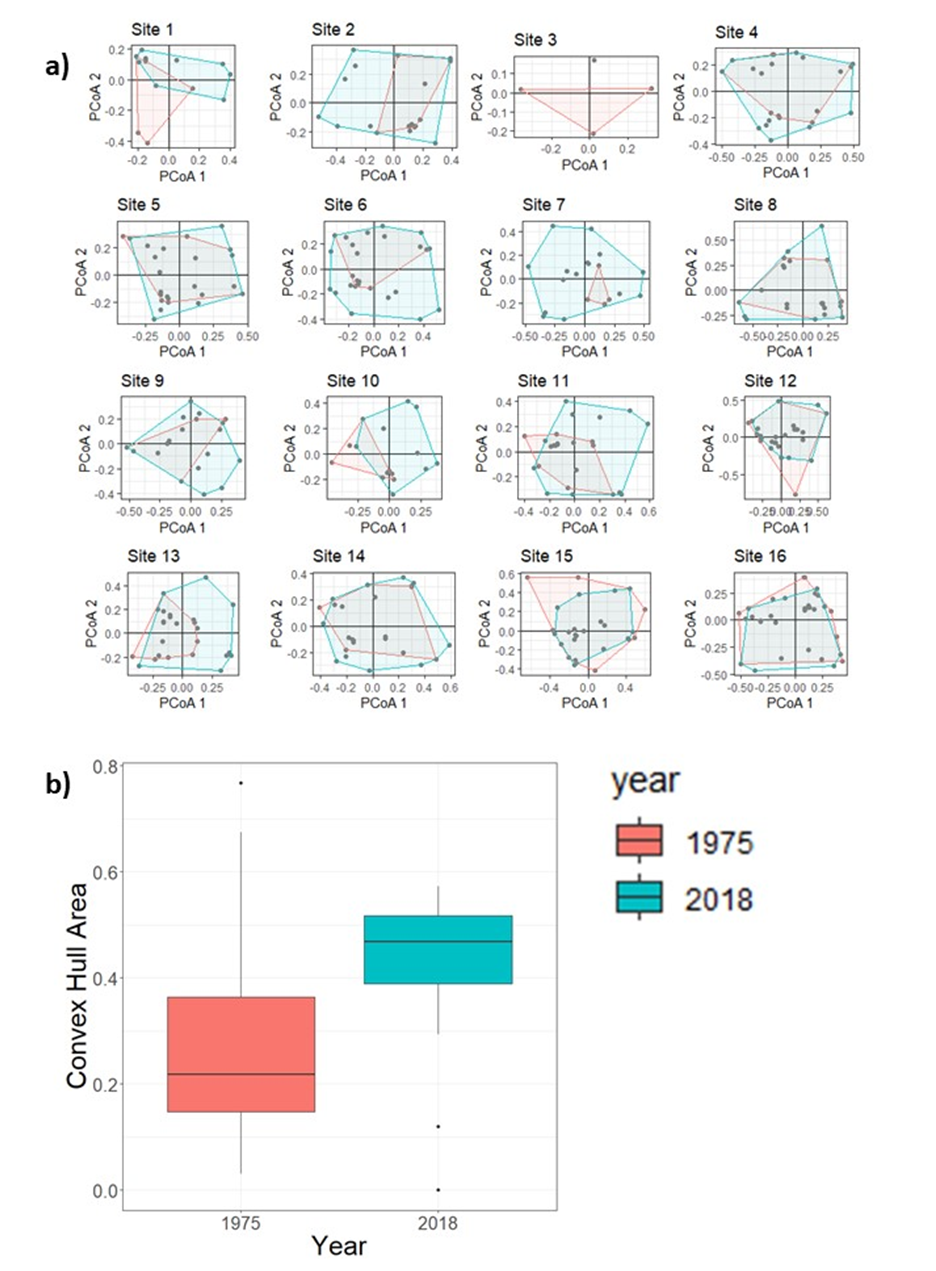
**Supplementary Fig. 5. a)** Gower distance-based principle coordinate analyses (PCoA) of coral traits present across each study site. Trait space for 1975-6 is represented by the pink polygon, and trait-space for 2018 is represented by the blue polygon. Grey dots represent individual coral genera. **b)** Boxplot of the site-based trait-space polygon hull areas for 1976-6 and 2018.


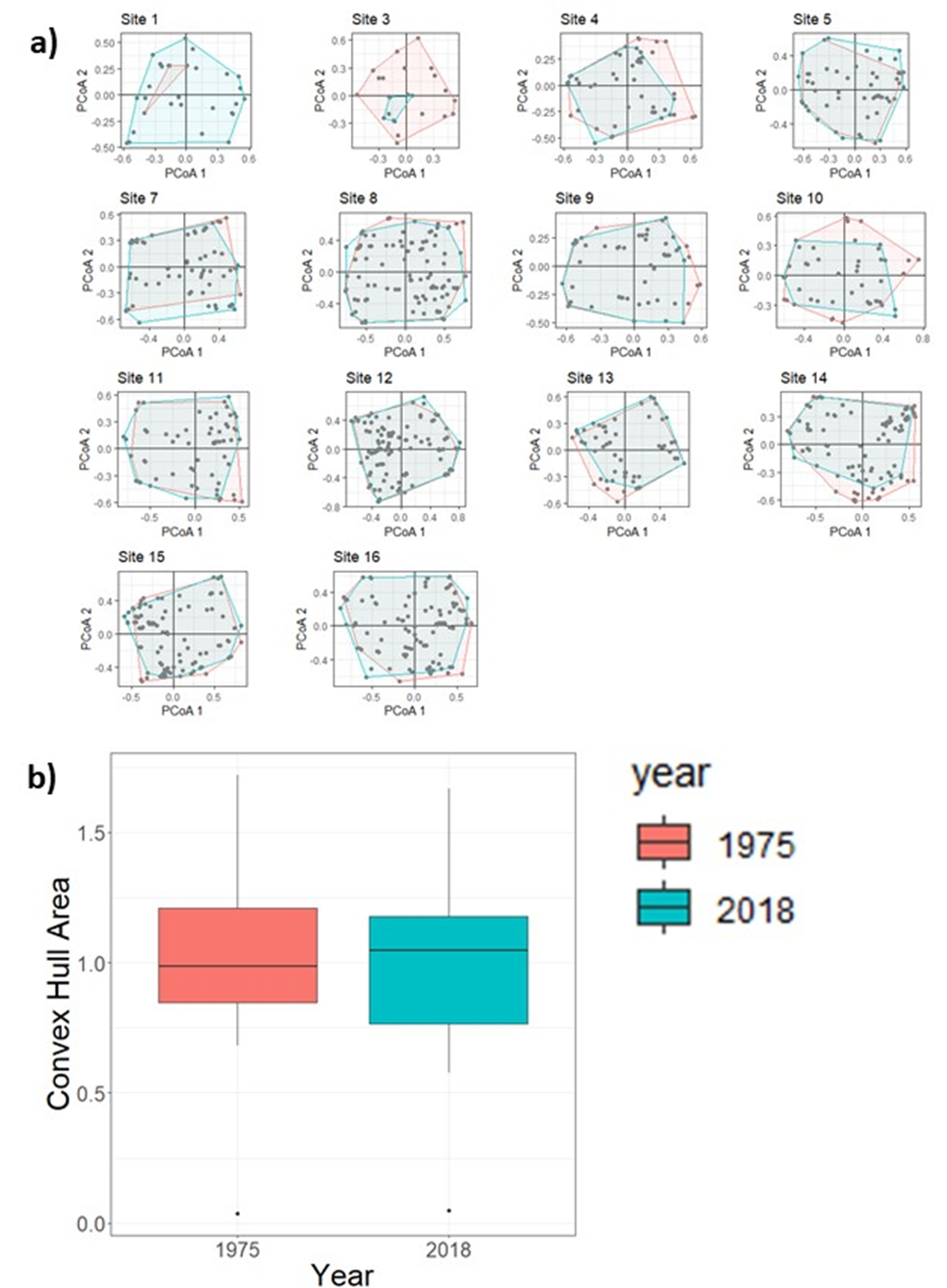


**Supplementary Fig. 6. a)** Gower distance-based principal coordinate analyses (PCoA) of fish traits present across each study site. Trait space for 1975-6 is represented by the pink polygon, and trait-space for 2018 is represented by the blue polygon. Grey dots represent individual fish species. b) Boxplot of the site-based trait-space polygon hull areas for 1976-6 and 2018.

**Supplementary Material Table S1.** Coral genera recorded in 1975-6 only, both 1975-6 and 2018, and 2018 only at 16 sites across Nakagusuku Bay.

| Recorded 1975-6 only | Recorded 1975-6 and 2018 | Recorded 2018 only |
| --- | --- | --- |
| *Coelastrea* | *Acropora* | *Alveopora* |
| *Cynarina* | *Astrea* | *Coscinaraea* |
| *Danafungia* | *Astreopora* | *Heteropsammia* |
| *Lithophyllon* | *Cyphastrea* | *Leptoria* |
| *Merulina* | *Dipsastraea* | *Leptoseris* |
| *Mycedium* | *Echinophyllia* | *Plerogyra* |
| *Sandalolitha* | *Echinopora* | *Symphyllia* |
|  | *Euphyllia* | *Trachyphyllia* |
|  | *Favites* |  |
|  | *Fungia* |  |
|  | *Fungiidae_family* |  |
|  | *Galaxea* |  |
|  | *Goniastrea* |  |
|  | *Goniopora* |  |
|  | *Hydnophora* |  |
|  | *Leptastrea* |  |
|  | *Lobophyllia* |  |
|  | *Montipora* |  |
|  | *Oulastrea* |  |
|  | *Oxypora* |  |
|  | *Pachyseris* |  |
|  | *Pavona* |  |
|  | *Pectinia* |  |
|  | *Platygyra* |  |
|  | *Pocillopora* |  |
|  | *Porites* |  |
|  | *Psammocora* |  |
|  | *Seriatopora* |  |
|  | *Stylocoeniella* |  |
|  | *Stylophora* |  |
|  | *Turbinaria* |  |

**Supplementary Material Table S2.** Reef fish species recorded in 1975-6 only, both 1975-6 and 2018, and 2018 only at 14 sites across Nakagusuku Bay.

| Recorded 1975-6 only | Recorded both years | Recorded 2018 only |
| --- | --- | --- |
| *Acanthurus bariene* | *Abudefduf sexfasciatus* | *Abudefduf septemfasciatus* |
| *Acanthurus triostegus* | *Abudefduf vaigiensis* | *Acanthurus blochii* |
| *Aeoliscus strigatus* | *Acanthurus lineatus* | *Acanthurus maculiceps* |
| *Amblygobius phalaena* | *Acanthurus olivaceus* | *Acanthurus nigricauda* |
| *Amphiprion clarkii* | *Amblyglyphidodon curacao* | *Acanthurus nigrofuscus* |
| *Amphiprion ocellaris* | *Amblyglyphidodon leucogaster* | *Anampses geographicus* |
| *Amphiprion perideraion* | *Amphiprion frenatus* | *Arothron meleagris* |
| *Amphiprion sandaracinos* | *Balistoides conspicillum* | *Arothron nigropunctatus* |
| *Anampses caeruleopunctatus* | *Bodianus loxozonus* | *Balistapus undulatus* |
| *Anampses meleagrides* | *Caesio caerulaurea* | *Balistoides viridescens* |
| *Atrosalarias holomelas* | *Caesio cuning* | *Bodianus izuensis* |
| *Aulostomus chinensis* | *Caesio teres* | *Calotomus japonicus* |
| *Bodianus axillaris* | *Canthigaster valentini* | *Caranx melampygus* |
| *Bodianus perditio* | *Centropyge ferrugata* | *Centropyge bispinosa* |
| *Canthigaster janthinoptera* | *Centropyge vrolikii* | *Cephalopholis argus* |
| *Centropyge bicolor* | *Chaetodon argentatus* | *Cephalopholis boenak* |
| *Centropyge tibicen* | *Chaetodon auriga* | *Cephalopholis leopardus* |
| *Cephalopholis urodeta* | *Chaetodon baronessa* | *Chaetodon auripes* |
| *Cetoscarus ocellatus* | *Chaetodon ephippium* | *Chaetodon guentheri* |
| *Chaetodon bennetti* | *Chaetodon kleinii* | *Cheilinus chlorourus* |
| *Chaetodon citrinellus* | *Chaetodon lunula* | *Cheilinus fasciatus* |
| *Chaetodon melannotus* | *Chaetodon lunulatus* | *Cheilio inermis* |
| *Chaetodon plebeius* | *Chaetodon ornatissimus* | *Cheilodipterus intermedius* |
| *Chaetodon reticulatus* | *Chaetodon speculum* | *Chlorurus microrhinos* |
| *Chaetodon ulietensis* | *Chaetodon vagabundus* | *Choerodon jordani* |
| *Chaetodon xanthurus* | *Chaetodontoplus mesoleucus* | *Chromis albicauda* |
| *Cheilodipterus macrodon* | *Cheilinus trilobatus* | *Chromis alleni* |
| *Cheilodipterus quinquelineatus* | *Chlorurus bowersi* | *Chromis delta* |
| *Cheiloprion labiatus* | *Chlorurus sordidus* | *Chromis notata* |
| *Chromis lepidolepis* | *Choerodon fasciatus* | *Chromis ovatiformes* |
| *Chromis ovatiformis* | *Choerodon schoenleinii* | *Chromis xanthura* |
| *Chromis* sp. | *Chromis atripes* | *Chrysiptera parasema* |
| *Chromis vanderbilti* | *Chromis chrysura* | *Chrysiptera starcki* |
| *Chromis viridis* | *Chromis flavomaculata* | *Chrysiptera unimaculata* |
| *Chromis weberi* | *Chromis fumea* | *Cirrhilabrus katherinae* |
| *Chromis xanthochira* | *Chromis margaritifer* | *Coris batuensis* |
| *Chrysiptera biocellata* | *Chromis ternatensis* | *Coris dorsomacula* |
| *Chrysiptera* sp*.* | *Chrysiptera cyanea* | *Coris gaimard* |
| *Cirrhilabrus temminckii* | *Chrysiptera glauca* | *Ctenochaetus binotatus* |
| *Cirrhitichthys aprinus* | *Chrysiptera rex* | *Ctenochaetus striatus* |
| *Cromileptes altivelis* | *Cirrhilabrus cyanopleura* | *Dascyllus trimaculatus* |
| *Ctenochaetus strigosus* | *Diploprion bifasciatum* | *Diagramma pictum* |
| *Dascyllus aruanus* | *Epinephelus merra* | *Dischistodus prosopotaenia* |
| *Dascyllus reticulatus* | *Forcipiger longirostris* | *Epibulus insidiator* |
| *Diademichthys lineatus* | *Gomphosus varius* | *Epinephelus polyphekadion* |
| *Ecsenius bicolor* | *Halichoeres prosopeion* | *Forcipiger flavissimus* |
| *Elagatis bipinnulata* | *Hemigymnus fasciatus* | *Gnathodentex aureolineatus* |
| *Epinephelus cyanopodus* | *Heniochus chrysostomus* | *Halichoeres chrysus* |
| *Epinephelus fasciatus* | *Labroides bicolor* | *Halichoeres nebulosus* |
| *Epinephelus quoyanus* | *Labroides dimidiatus* | *Halichoeres scapularis* |
| *Fistularia commersonii* | *Lutjanus vitta* | *Heniochus varius* |
| *Halichoeres hortulanus* | *Meiacanthus kamoharai* | *Labrichthys unilineatus* |
| *Halichoeres leucurus* | *Naso lituratus* | *Lutjanus bohar* |
| *Halichoeres melanochir* | *Nemateleotris magnifica* | *Lutjanus fulviflamma* |
| *Halichoeres trimaculatus* | *Neoglyphidodon melas* | *Lutjanus gibbus* |
| *Hemigymnus melapterus* | *Neoglyphidodon nigroris* | *Lutjanus lutjanus* |
| *Heniochus acuminatus* | *Paraluteres prionurus* | *Lutjanus quinquelineatus* |
| *Heniochus monoceros* | *Parapercis pacifica* | *Macolor niger* |
| *Heniochus singularius* | *Parupeneus barberinoides* | *Mulloidichthys vanicolensis* |
| *Iniistius dea* | *Plagiotremus tapeinosoma* | *Myripristis hexagona* |
| *Koumansetta hectori* | *Plectorhinchus chaetodonoides* | *Naso hexacanthus* |
| *Labracinus* sp. | *Plectropomus leopardus* | *Neoniphon sammara* |
| *Labropsis manabei* | *Pomacanthus semicirculatus* | *Neopomacentrus cyanomos* |
| *Lethrinus nebulosus* | *Pomacentrus bankanensis* | *Neopomacentrus violascens* |
| *Lutjanus kasmira* | *Pomacentrus brachialis* | *Oplegnathus punctatus* |
| *Meiacanthus* sp*.* | *Pomacentrus chrysurus* | *Ostorhinchus angustatus* |
| *Myripristis murdjan* | *Pomacentrus coelestis* | *Ostorhinchus endekataenia* |
| *Naso brevirostris* | *Pomacentrus lepidogenys* | *Paracaesio xanthura* |
| *Nematalosa japonica* | *Pomacentrus moluccensis* | *Parapercis clathrata* |
| *Ostorhinchus ishigakiensis* | *Pomacentrus nagasakiensis* | *Parapercis hexophtalma* |
| *Ostorhinchus properuptus* | *Pomacentrus philippinus* | *Parupeneus barberinus* |
| *Ostracion cubicus* | *Pomachromis richardsoni* | *Parupeneus multifasciatus* |
| *Ostracion meleagris* | *Ptereleotris evides* | *Parupeneus spilurus* |
| *Oxymonacanthus longirostris* | *Pygoplites diacanthus* | *Plagiotremus rhinorhynchos* |
| *Paracirrhites arcatus* | *Sargocentron rubrum* | *Plectorhinchus lessonii* |
| *Paracirrhites forsteri* | *Scarus ghobban* | *Pomacanthus sexstriatus* |
| *Parapercis cylindrica* | *Scolopsis bilineata* | *Pomacentrus alexanderae* |
| *Parupeneus crassilabris* | *Siganus argenteus* | *Pomacentrus amboinensis* |
| *Parupeneus cyclostomus* | *Siganus virgatus* | *Pomacentrus nigromarginatus* |
| *Parupeneus indicus* | *Sufflamen chrysopterum* | *Pomacentrus vaiuli* |
| *Pervagor melanocephalus* | *Symphorus nematophorus* | *Prionurus scalprum* |
| *Plagiotremus laudandus* | *Thalassoma hardwicke* | *Pseudodax moluccanus* |
| *Platax pinnatus* | *Thalassoma lunare* | *Pseudojuloides elongatus* |
| *Plectroglyphidodon dickii* | *Thalassoma lutescens* | *Ptereleotris microlepis* |
| *Plectroglyphidodon lacrymatus* | *Zanclus cornutus* | *Pterocaesio marri* |
| *Pomacanthus imperator* | *Zebrasoma scopas* | *Rhinecanthus rectangulus* |
| *Pomacentrus* sp. | *Zebrasoma velifer* | *Sargocentron spinosissimum* |
| *Pomachromis* sp. |  | *Scarus chameleon* |
| *Pseudocaranx dentex* |  | *Scarus festivus* |
| *Pseudocheilinus hexataenia* |  | *Scarus forsteni* |
| *Pterocaesio tile* |  | *Scarus fuscocaudalis* |
| *Pterois lunulata* |  | *Scarus globiceps* |
| *Rhinecanthus aculeatus* |  | *Scarus hypselopterus* |
| *Sargocentron spiniferum* |  | *Scarus ovifrons* |
| *Scarus prasiognathos* |  | *Scarus rivulatus* |
| *Scarus scaber* |  | *Scarus rubroviolaceus* |
| *Scarus* sp. |  | *Scarus schlegeli* |
| *Siganus puellus* |  | *Scolopsis affinis* |
| *Siganus unimaculatus* |  | *Scolopsis lineata* |
| *Stethojulis interrupta* |  | *Scolopsis monogramma* |
| *Synodus variegatus* |  | *Stegastes altus* |
| *Syphraena* sp*.* |  | *Stegastes fasciolatus* |
| *Thalassoma amblycephalum* |  | *Stegastes nigricans* |
| *Thalassoma jansenii* |  | *Stegastes obreptus* |
| *Thalassoma trilobatum* |  | *Stegastes punctatus* |
| *Trachyrhamphus serratus* |  | *Stethojulis trilineata* |
| *Zebrasoma flavescens* |  | *Thalassoma quinquevittatum* |
|  |  | *Upeneus tragula* |

**Supplementary Material Table S3.** SIMPER analyses of average presence/ absence of coral genera contributing to differences (Bray-Curtis distance) between 1975-6 and 2018 at 16 sites across Nakagusuku Bay.

| Genera | Mean 1975-6 | Mean 2018 | Consistency ratio | Cumulative contribution |
| --- | --- | --- | --- | --- |
| *Turbinaria* | 0.063 | 0.867 | 1.825 | 0.052 |
| *Astreopora* | 0.250 | 1.000 | 1.534 | 0.101 |
| *Psammocora* | 0.250 | 0.867 | 1.325 | 0.146 |
| *Astrea* | 0.063 | 0.733 | 1.422 | 0.189 |
| *Pavona* | 0.250 | 0.733 | 1.188 | 0.229 |
| *Favites* | 0.438 | 0.933 | 1.069 | 0.267 |
| *Galaxea* | 0.375 | 0.800 | 1.130 | 0.304 |
| *Trachyphyllia* | 0.000 | 0.600 | 1.151 | 0.341 |
| *Pachyseris* | 0.250 | 0.667 | 1.144 | 0.377 |
| *Lobophyllia* | 0.125 | 0.600 | 1.117 | 0.411 |
| *Stylophora* | 0.563 | 0.467 | 0.942 | 0.444 |
| *Leptastrea* | 0.188 | 0.533 | 0.987 | 0.476 |
| *Goniastrea* | 0.563 | 1.000 | 0.849 | 0.508 |
| *Seriatopora* | 0.438 | 0.533 | 0.962 | 0.540 |
| *Pocillopora* | 0.563 | 0.667 | 0.924 | 0.572 |
| *Dipsastraea* | 0.563 | 1.000 | 0.843 | 0.603 |
| *Platygyra* | 0.375 | 0.400 | 0.898 | 0.632 |
| *Hydnophora* | 0.313 | 0.400 | 0.869 | 0.662 |
| Fungiidae_family | 0.125 | 0.467 | 0.923 | 0.689 |
| *Cyphastrea* | 0.625 | 1.000 | 0.746 | 0.717 |
| *Coscinaraea* | 0.000 | 0.400 | 0.791 | 0.741 |
| *Montipora* | 0.750 | 0.800 | 0.699 | 0.764 |
| *Stylocoeniella* | 0.313 | 0.067 | 0.677 | 0.783 |
| *Lithophyllon* | 0.375 | 0.000 | 0.757 | 0.803 |
| *Oulastrea* | 0.063 | 0.267 | 0.627 | 0.821 |
| *Pectinia* | 0.188 | 0.200 | 0.660 | 0.838 |
| *Leptoria* | 0.000 | 0.267 | 0.587 | 0.854 |
| *Oxypora* | 0.063 | 0.200 | 0.542 | 0.868 |
| *Fungia* | 0.125 | 0.133 | 0.521 | 0.881 |
| *Echinopora* | 0.125 | 0.133 | 0.531 | 0.893 |
| *Symphyllia* | 0.000 | 0.200 | 0.488 | 0.906 |
| *Echinophyllia* | 0.188 | 0.067 | 0.537 | 0.917 |
| *Leptoseris* | 0.000 | 0.200 | 0.491 | 0.928 |
| *Goniopora* | 0.063 | 0.133 | 0.453 | 0.939 |
| *Coelastrea* | 0.125 | 0.000 | 0.369 | 0.948 |
| *Danafungia* | 0.125 | 0.000 | 0.372 | 0.955 |
| *Euphyllia* | 0.063 | 0.067 | 0.359 | 0.961 |
| *Merulina* | 0.125 | 0.000 | 0.375 | 0.967 |
| *Mycedium* | 0.125 | 0.000 | 0.375 | 0.973 |
| *Acropora* | 0.938 | 1.000 | 0.252 | 0.978 |
| *Porites* | 0.938 | 1.000 | 0.252 | 0.983 |
| *Plerogyra* | 0.000 | 0.067 | 0.262 | 0.987 |
| *Alveopora* | 0.000 | 0.067 | 0.263 | 0.991 |
| *Heteropsammia* | 0.000 | 0.067 | 0.263 | 0.994 |
| *Sandalolitha* | 0.063 | 0.000 | 0.256 | 0.997 |
| *Cynarina* | 0.063 | 0.000 | 0.257 | 1.000 |

**Supplementary Material Table S4.** SIMPER analyses of average presence/ absence of fish species contributing to differences (Bray-Curtis distance) between 1975-6 and 2018 at 14 sites across Nakagusuku Bay.

| Species | Mean 1975-6 | Mean 2018 | | Consistency ratio | Cumulative contribution |
| --- | --- | --- | --- | --- | --- |
| *Acanthurus nigrofuscus* | 0.000 | 0.857 | 1.632 | | 0.014 |
| *Ctenochaetus binotatus* | 0.000 | 0.714 | 1.221 | | 0.025 |
| *Sargocentron rubrum* | 0.714 | 0.071 | 1.152 | | 0.036 |
| *Meiacanthus* sp. | 0.714 | 0.000 | 1.331 | | 0.047 |
| *Chaetodon plebeius* | 0.714 | 0.000 | 1.252 | | 0.058 |
| *Siganus virgatus* | 0.286 | 0.786 | 1.087 | | 0.069 |
| *Ctenochaetus strigosus* | 0.643 | 0.000 | 0.719 | | 0.079 |
| *Abudefduf sexfasciatus* | 0.214 | 0.714 | 1.066 | | 0.090 |
| *Chrysiptera rex* | 0.357 | 0.714 | 0.928 | | 0.100 |
| *Thalassoma lutescens* | 0.786 | 0.357 | 1.014 | | 0.110 |
| *Scarus rivulatus* | 0.000 | 0.643 | 1.147 | | 0.120 |
| *Stegastes altus* | 0.000 | 0.643 | 1.144 | | 0.129 |
| *Chrysiptera cyanea* | 0.286 | 0.571 | 0.878 | | 0.139 |
| *Meiacanthus kamoharai* | 0.500 | 0.214 | 0.883 | | 0.148 |
| *Halichoeres prosopeion* | 0.500 | 0.143 | 0.610 | | 0.158 |
| *Parupeneus multifasciatus* | 0.000 | 0.571 | 1.011 | | 0.167 |
| *Zebrasoma velifer* | 0.214 | 0.571 | 0.947 | | 0.176 |
| *Sufflamen chrysopterum* | 0.571 | 0.429 | 0.846 | | 0.184 |
| *Labroides dimidiatus* | 0.571 | 0.714 | 0.822 | | 0.193 |
| *Scolopsis bilineata* | 0.286 | 0.500 | 0.859 | | 0.201 |
| *Coris batuensis* | 0.000 | 0.429 | 0.545 | | 0.210 |
| *Pomacentrus moluccensis* | 0.714 | 0.571 | 0.766 | | 0.218 |
| *Chromis margaritifer* | 0.429 | 0.500 | 0.889 | | 0.226 |
| *Pomacentrus coelestis* | 0.286 | 0.429 | 0.804 | | 0.234 |
| *Chaetodon auriga* | 0.143 | 0.357 | 0.517 | | 0.242 |
| *Chaetodon citrinellus* | 0.500 | 0.000 | 0.867 | | 0.250 |
| *Pomacentrus brachialis* | 0.500 | 0.143 | 0.855 | | 0.258 |
| *Zanclus cornutus* | 0.714 | 0.714 | 0.704 | | 0.266 |
| *Pomacentrus lepidogenys* | 0.500 | 0.429 | 0.922 | | 0.274 |
| *Chlorurus sordidus* | 0.143 | 0.500 | 0.894 | | 0.281 |
| *Chlorurus bowersi* | 0.071 | 0.500 | 0.900 | | 0.289 |
| *Epinephelus fasciatus* | 0.500 | 0.000 | 0.904 | | 0.296 |
| *Chaetodon lunulatus* | 0.786 | 0.714 | 0.691 | | 0.304 |
| *Pomacentrus nagasakiensis* | 0.357 | 0.214 | 0.751 | | 0.311 |
| *Chromis chrysura* | 0.500 | 0.214 | 0.920 | | 0.319 |
| *Amphiprion frenatus* | 0.429 | 0.214 | 0.790 | | 0.326 |
| *Choerodon schoenleinii* | 0.143 | 0.357 | 0.702 | | 0.333 |
| *Amblyglyphidodon curacao* | 0.357 | 0.357 | 0.820 | | 0.340 |
| *Parupeneus barberinoides* | 0.071 | 0.357 | 0.678 | | 0.347 |
| *Plagiotremus laudandus* | 0.500 | 0.000 | 0.893 | | 0.354 |
| *Neoglyphidodon nigroris* | 0.429 | 0.071 | 0.807 | | 0.361 |
| *Plagiotremus rhinorhynchos* | 0.000 | 0.357 | 0.654 | | 0.368 |
| *Thalassoma lunare* | 0.429 | 0.071 | 0.761 | | 0.375 |
| *Chaetodon vagabundus* | 0.286 | 0.214 | 0.684 | | 0.382 |
| *Parapercis pacifica* | 0.357 | 0.071 | 0.691 | | 0.388 |
| *Pomachromis richardsoni* | 0.357 | 0.286 | 0.766 | | 0.395 |
| *Cheilodipterus quinquelineatus* | 0.429 | 0.000 | 0.738 | | 0.402 |
| *Pomacentrus alexanderae* | 0.000 | 0.429 | 0.776 | | 0.408 |
| *Canthigaster valentini* | 0.429 | 0.143 | 0.815 | | 0.414 |
| *Gomphosus varius* | 0.357 | 0.286 | 0.813 | | 0.421 |
| *Scarus ghobban* | 0.286 | 0.286 | 0.748 | | 0.427 |
| *Abudefduf vaigiensis* | 0.286 | 0.286 | 0.761 | | 0.433 |
| *Hemigymnus fasciatus* | 0.357 | 0.286 | 0.820 | | 0.439 |
| *Centropyge vrolikii* | 0.357 | 0.214 | 0.761 | | 0.445 |
| *Epinephelus merra* | 0.357 | 0.214 | 0.771 | | 0.451 |
| *Oxymonacanthus longirostris* | 0.429 | 0.000 | 0.787 | | 0.457 |
| *Lutjanus fulviflamma* | 0.000 | 0.286 | 0.393 | | 0.463 |
| *Acanthurus lineatus* | 0.357 | 0.214 | 0.763 | | 0.469 |
| *Zebrasoma scopas* | 0.286 | 0.286 | 0.762 | | 0.475 |
| *Cheilodipterus macrodon* | 0.357 | 0.000 | 0.656 | | 0.481 |
| *Pomacentrus philippinus* | 0.357 | 0.214 | 0.799 | | 0.486 |
| *Thalassoma hardwicke* | 0.286 | 0.143 | 0.632 | | 0.492 |
| *Pomacentrus chrysurus* | 0.286 | 0.071 | 0.616 | | 0.497 |
| *Pseudocheilinus hexataenia* | 0.357 | 0.000 | 0.647 | | 0.502 |
| *Chaetodon argentatus* | 0.357 | 0.143 | 0.730 | | 0.508 |
| *Amblyglyphidodon leucogaster* | 0.357 | 0.143 | 0.726 | | 0.513 |
| *Caesio teres* | 0.071 | 0.286 | 0.609 | | 0.518 |
| *Scarus scaber* | 0.429 | 0.000 | 0.807 | | 0.524 |
| *Chaetodon kleinii* | 0.286 | 0.143 | 0.671 | | 0.529 |
| *Heniochus monoceros* | 0.357 | 0.000 | 0.646 | | 0.534 |
| *Scarus* sp. | 0.214 | 0.000 | 0.333 | | 0.539 |
| *Epinephelus cyanopodus* | 0.357 | 0.000 | 0.686 | | 0.544 |
| *Halichoeres trimaculatus* | 0.357 | 0.000 | 0.676 | | 0.549 |
| *Amphiprion clarkii* | 0.357 | 0.000 | 0.674 | | 0.554 |
| *Pomacanthus semicirculatus* | 0.071 | 0.286 | 0.616 | | 0.558 |
| *Parupeneus barberinus* | 0.000 | 0.286 | 0.536 | | 0.563 |
| *Synodus variegatus* | 0.286 | 0.000 | 0.587 | | 0.568 |
| *Rhinecanthus aculeatus* | 0.286 | 0.000 | 0.595 | | 0.572 |
| *Halichoeres hortulanus* | 0.286 | 0.000 | 0.546 | | 0.577 |
| *Naso brevirostris* | 0.286 | 0.000 | 0.556 | | 0.581 |
| *Ostorhinchus sp.* | 0.286 | 0.000 | 0.574 | | 0.585 |
| *Diademichthys lineatus* | 0.214 | 0.000 | 0.488 | | 0.590 |
| *Ostracion cubicus* | 0.286 | 0.000 | 0.573 | | 0.594 |
| *Pervagor melanocephalus* | 0.286 | 0.000 | 0.592 | | 0.598 |
| *Bodianus loxozonus* | 0.286 | 0.143 | 0.710 | | 0.602 |
| *Labroides bicolor* | 0.286 | 0.143 | 0.710 | | 0.606 |
| *Heniochus chrysostomus* | 0.286 | 0.071 | 0.635 | | 0.610 |
| *Scarus schlegeli* | 0.000 | 0.286 | 0.570 | | 0.614 |
| *Centropyge tibicen* | 0.357 | 0.000 | 0.727 | | 0.618 |
| *Parupeneus spilurus* | 0.000 | 0.214 | 0.473 | | 0.622 |
| *Cheilinus trilobatus* | 0.071 | 0.214 | 0.506 | | 0.626 |
| *Plagiotremus tapeinosoma* | 0.214 | 0.071 | 0.520 | | 0.630 |
| *Chromis ternatensis* | 0.286 | 0.071 | 0.539 | | 0.633 |
| *Halichoeres scapularis* | 0.000 | 0.143 | 0.260 | | 0.637 |
| *Neoglyphidodon melas* | 0.143 | 0.143 | 0.528 | | 0.641 |
| *Forcipiger longirostris* | 0.286 | 0.071 | 0.661 | | 0.645 |
| *Chromis xanthura* | 0.000 | 0.214 | 0.468 | | 0.648 |
| *Ostorhinchus properuptus* | 0.214 | 0.000 | 0.456 | | 0.652 |
| *Caesio caerulaurea* | 0.143 | 0.071 | 0.450 | | 0.655 |
| *Parapercis clathrata* | 0.000 | 0.214 | 0.481 | | 0.659 |
| *Stegastes obreptus* | 0.000 | 0.214 | 0.482 | | 0.663 |
| *Chromis viridis* | 0.286 | 0.000 | 0.586 | | 0.666 |
| *Plectroglyphidodon dickii* | 0.286 | 0.000 | 0.586 | | 0.670 |
| *Halichoeres chrysus* | 0.000 | 0.214 | 0.486 | | 0.673 |
| *Chrysiptera glauca* | 0.071 | 0.143 | 0.426 | | 0.677 |
| *Naso lituratus* | 0.071 | 0.214 | 0.546 | | 0.680 |
| *Chaetodon speculum* | 0.214 | 0.071 | 0.546 | | 0.683 |
| *Cheilinus chlorourus* | 0.000 | 0.214 | 0.491 | | 0.687 |
| *Aulostomus chinensis* | 0.214 | 0.000 | 0.461 | | 0.690 |
| *Fistularia commersonii* | 0.214 | 0.000 | 0.461 | | 0.693 |
| *Paraluteres prionurus* | 0.143 | 0.143 | 0.535 | | 0.696 |
| *Diploprion bifasciatum* | 0.214 | 0.071 | 0.546 | | 0.700 |
| *Chaetodon reticulatus* | 0.214 | 0.000 | 0.449 | | 0.703 |
| *Scarus prasiognathos* | 0.286 | 0.000 | 0.618 | | 0.706 |
| *Balistoides viridescens* | 0.000 | 0.214 | 0.494 | | 0.709 |
| *Caesio cuning* | 0.143 | 0.143 | 0.545 | | 0.712 |
| *Halichoeres nebulosus* | 0.000 | 0.143 | 0.365 | | 0.715 |
| *Pomacentrus amboinensis* | 0.000 | 0.143 | 0.365 | | 0.718 |
| *Chaetodon baronessa* | 0.214 | 0.071 | 0.564 | | 0.721 |
| *Siganus argenteus* | 0.143 | 0.071 | 0.462 | | 0.724 |
| *Choerodon fasciatus* | 0.143 | 0.143 | 0.555 | | 0.727 |
| *Balistoides conspicillum* | 0.071 | 0.143 | 0.462 | | 0.730 |
| *Chromis atripes* | 0.214 | 0.071 | 0.572 | | 0.733 |
| *Myripristis murdjan* | 0.214 | 0.000 | 0.475 | | 0.736 |
| *Ctenochaetus striatus* | 0.000 | 0.214 | 0.491 | | 0.739 |
| *Scarus chameleon* | 0.000 | 0.214 | 0.491 | | 0.741 |
| *Cirrhilabrus cyanopleura* | 0.143 | 0.143 | 0.553 | | 0.744 |
| *Chromis flavomaculata* | 0.214 | 0.071 | 0.573 | | 0.747 |
| *Chromis alleni* | 0.000 | 0.214 | 0.491 | | 0.750 |
| *Acanthurus olivaceus* | 0.143 | 0.071 | 0.464 | | 0.753 |
| *Chromis fumea* | 0.071 | 0.143 | 0.463 | | 0.755 |
| *Labropsis manabei* | 0.214 | 0.000 | 0.486 | | 0.758 |
| *Plectropomus leopardus* | 0.143 | 0.071 | 0.456 | | 0.761 |
| *Ptereleotris evides* | 0.143 | 0.071 | 0.457 | | 0.763 |
| *Chaetodon ephippium* | 0.071 | 0.071 | 0.357 | | 0.766 |
| *Epinephelus quoyanus* | 0.143 | 0.000 | 0.392 | | 0.768 |
| *Chaetodontoplus mesoleucus* | 0.143 | 0.071 | 0.462 | | 0.771 |
| *Parapercis hexophtalma* | 0.000 | 0.143 | 0.376 | | 0.773 |
| *Dischistodus prosopotaenia* | 0.000 | 0.143 | 0.376 | | 0.776 |
| *Siganus puellus* | 0.214 | 0.000 | 0.513 | | 0.778 |
| *Siganus unimaculatus* | 0.214 | 0.000 | 0.513 | | 0.781 |
| *Chaetodon lunula* | 0.143 | 0.071 | 0.460 | | 0.783 |
| *Dascyllus trimaculatus* | 0.000 | 0.143 | 0.346 | | 0.785 |
| *Pomacentrus vaiuli* | 0.000 | 0.143 | 0.363 | | 0.788 |
| *Bodianus perditio* | 0.214 | 0.000 | 0.510 | | 0.790 |
| *Cirrhilabrus temminckii* | 0.214 | 0.000 | 0.510 | | 0.792 |
| *Paracirrhites arcatus* | 0.214 | 0.000 | 0.510 | | 0.795 |
| *Paracirrhites forsteri* | 0.214 | 0.000 | 0.510 | | 0.797 |
| *Symphorus nematophorus* | 0.071 | 0.071 | 0.334 | | 0.799 |
| *Amphiprion perideraion* | 0.214 | 0.000 | 0.512 | | 0.802 |
| *Pomacentrus bankanensis* | 0.143 | 0.071 | 0.481 | | 0.804 |
| *Cephalopholis boenak* | 0.000 | 0.143 | 0.357 | | 0.806 |
| *Chaetodon guentheri* | 0.000 | 0.143 | 0.385 | | 0.808 |
| *Lutjanus quinquelineatus* | 0.000 | 0.143 | 0.386 | | 0.811 |
| *Thalassoma jansenii* | 0.143 | 0.000 | 0.383 | | 0.813 |
| *Chromis albicauda* | 0.000 | 0.143 | 0.387 | | 0.815 |
| *Zebrasoma flavescens* | 0.143 | 0.000 | 0.386 | | 0.817 |
| *Chromis xanthochira* | 0.143 | 0.000 | 0.387 | | 0.819 |
| *Pygoplites diacanthus* | 0.143 | 0.071 | 0.481 | | 0.821 |
| *Arothron meleagris* | 0.000 | 0.143 | 0.387 | | 0.823 |
| *Scarus hypselopterus* | 0.000 | 0.143 | 0.387 | | 0.825 |
| *Stegastes nigricans* | 0.000 | 0.143 | 0.387 | | 0.827 |
| *Cetoscarus ocellatus* | 0.143 | 0.000 | 0.383 | | 0.830 |
| *Acanthurus triostegus* | 0.143 | 0.000 | 0.384 | | 0.832 |
| *Gnathodentex aureolineatus* | 0.000 | 0.143 | 0.387 | | 0.834 |
| *Chaetodon ulietensis* | 0.143 | 0.000 | 0.375 | | 0.835 |
| *Plectorhinchus chaetodonoides* | 0.071 | 0.071 | 0.342 | | 0.837 |
| *Coris dorsomacula* | 0.000 | 0.143 | 0.389 | | 0.839 |
| *Chaetodon bennetti* | 0.143 | 0.000 | 0.376 | | 0.841 |
| *Anampses caeruleopunctatus* | 0.143 | 0.000 | 0.377 | | 0.843 |
| *Chromis lepidolepis* | 0.143 | 0.000 | 0.377 | | 0.845 |
| *Dascyllus aruanus* | 0.143 | 0.000 | 0.377 | | 0.847 |
| *Epibulus insidiator* | 0.000 | 0.143 | 0.381 | | 0.849 |
| *Cephalopholis argus* | 0.000 | 0.143 | 0.383 | | 0.851 |
| *Chromis ovatiformes* | 0.000 | 0.143 | 0.386 | | 0.853 |
| *Pomacentrus nigromarginatus* | 0.000 | 0.143 | 0.386 | | 0.854 |
| *Cirrhilabrus katherinae* | 0.000 | 0.143 | 0.391 | | 0.856 |
| *Forcipiger flavissimus* | 0.000 | 0.143 | 0.394 | | 0.858 |
| *Scarus forsteni* | 0.000 | 0.143 | 0.394 | | 0.860 |
| *Ecsenius bicolor* | 0.143 | 0.000 | 0.401 | | 0.861 |
| *Koumansetta hectori* | 0.143 | 0.000 | 0.401 | | 0.863 |
| *Centropyge ferrugata* | 0.071 | 0.071 | 0.381 | | 0.865 |
| *Nemateleotris magnifica* | 0.071 | 0.071 | 0.381 | | 0.866 |
| *Canthigaster janthinoptera* | 0.143 | 0.000 | 0.401 | | 0.868 |
| *Aeoliscus strigatus* | 0.071 | 0.000 | 0.260 | | 0.869 |
| *Amblygobius phalaena* | 0.071 | 0.000 | 0.260 | | 0.871 |
| *Heniochus acuminatus* | 0.071 | 0.000 | 0.260 | | 0.873 |
| *Ostorhinchus ishigakiensis* | 0.071 | 0.000 | 0.260 | | 0.874 |
| *Trachyrhamphus serratus* | 0.071 | 0.000 | 0.260 | | 0.876 |
| *Parupeneus cyclostomus* | 0.143 | 0.000 | 0.401 | | 0.878 |
| *Neoniphon sammara* | 0.000 | 0.071 | 0.248 | | 0.879 |
| *Chaetodon melannotus* | 0.143 | 0.000 | 0.402 | | 0.881 |
| *Chaetodon xanthurus* | 0.143 | 0.000 | 0.402 | | 0.882 |
| *Chaetodon ornatissimus* | 0.071 | 0.071 | 0.383 | | 0.884 |
| *Lutjanus vitta* | 0.071 | 0.071 | 0.383 | | 0.885 |
| *Chromis ovatiformis* | 0.143 | 0.000 | 0.400 | | 0.887 |
| *Halichoeres leucurus* | 0.143 | 0.000 | 0.400 | | 0.888 |
| *Hemigymnus melapterus* | 0.143 | 0.000 | 0.400 | | 0.890 |
| *Platax pinnatus* | 0.143 | 0.000 | 0.400 | | 0.891 |
| *Plectroglyphidodon lacrymatus* | 0.143 | 0.000 | 0.400 | | 0.893 |
| *Bodianus izuensis* | 0.000 | 0.071 | 0.253 | | 0.894 |
| *Choerodon jordani* | 0.000 | 0.071 | 0.253 | | 0.896 |
| *Anampses meleagrides* | 0.143 | 0.000 | 0.402 | | 0.897 |
| *Stegastes punctatus* | 0.000 | 0.071 | 0.256 | | 0.899 |
| *Upeneus tragula* | 0.000 | 0.071 | 0.256 | | 0.900 |
| *Amblygobius* sp. | 0.071 | 0.000 | 0.265 | | 0.901 |
| *Chrysiptera* sp. | 0.071 | 0.000 | 0.265 | | 0.903 |
| *Rhinecanthus verrucosus* | 0.071 | 0.000 | 0.265 | | 0.904 |
| *Chlorurus microrhinos* | 0.000 | 0.071 | 0.256 | | 0.905 |
| *Oplegnathus punctatus* | 0.000 | 0.071 | 0.256 | | 0.907 |
| *Ptereleotris microlepis* | 0.000 | 0.071 | 0.256 | | 0.908 |
| *Pterois lunulata* | 0.071 | 0.000 | 0.266 | | 0.909 |
| *Arothron nigropunctatus* | 0.000 | 0.071 | 0.259 | | 0.911 |
| *Cheilinus fasciatus* | 0.000 | 0.071 | 0.259 | | 0.912 |
| *Cheilio inermis* | 0.000 | 0.071 | 0.259 | | 0.913 |
| *Iniistius dea* | 0.071 | 0.000 | 0.267 | | 0.914 |
| *Nematalosa japonica* | 0.071 | 0.000 | 0.267 | | 0.916 |
| *Halichoeres melanochir* | 0.071 | 0.000 | 0.267 | | 0.917 |
| *Acanthurus bariene* | 0.071 | 0.000 | 0.268 | | 0.918 |
| *Chrysiptera biocellata* | 0.071 | 0.000 | 0.268 | | 0.919 |
| *Lutjanus kasmira* | 0.071 | 0.000 | 0.268 | | 0.921 |
| *Parapercis cylindrica* | 0.071 | 0.000 | 0.268 | | 0.922 |
| *Pseudocaranx dentex* | 0.071 | 0.000 | 0.268 | | 0.923 |
| *Labrichthys unilineatus* | 0.000 | 0.071 | 0.263 | | 0.924 |
| *Ostorhinchus angustatus* | 0.000 | 0.071 | 0.263 | | 0.925 |
| *Pomacanthus sexstriatus* | 0.000 | 0.071 | 0.263 | | 0.927 |
| *Acanthurus nigricauda* | 0.000 | 0.071 | 0.263 | | 0.928 |
| *Balistapus undulatus* | 0.000 | 0.071 | 0.263 | | 0.929 |
| *Naso hexacanthus* | 0.000 | 0.071 | 0.263 | | 0.930 |
| *Prionurus scalprum* | 0.000 | 0.071 | 0.263 | | 0.931 |
| *Scarus festivus* | 0.000 | 0.071 | 0.263 | | 0.932 |
| *Stethojulis trilineata* | 0.000 | 0.071 | 0.263 | | 0.933 |
| *Thalassoma quinquevittatum* | 0.000 | 0.071 | 0.263 | | 0.934 |
| *Cephalopholis leopardus* | 0.000 | 0.071 | 0.263 | | 0.935 |
| *Lutjanus lutjanus* | 0.000 | 0.071 | 0.263 | | 0.937 |
| *Neopomacentrus cyanomos* | 0.000 | 0.071 | 0.263 | | 0.938 |
| *Ostorhinchus endekataenia* | 0.000 | 0.071 | 0.263 | | 0.939 |
| *Pseudodax moluccanus* | 0.000 | 0.071 | 0.263 | | 0.940 |
| *Sargocentron spinosissimum* | 0.000 | 0.071 | 0.263 | | 0.941 |
| *Chromis notata* | 0.000 | 0.071 | 0.265 | | 0.942 |
| *Chrysiptera parasema* | 0.000 | 0.071 | 0.265 | | 0.943 |
| *Epinephelus polyphekadion* | 0.000 | 0.071 | 0.265 | | 0.944 |
| *Neopomacentrus violascens* | 0.000 | 0.071 | 0.265 | | 0.945 |
| *Scarus ovifrons* | 0.000 | 0.071 | 0.265 | | 0.946 |
| *Scolopsis monogramma* | 0.000 | 0.071 | 0.265 | | 0.947 |
| *Abudefduf septemfasciatus* | 0.000 | 0.071 | 0.266 | | 0.948 |
| *Acanthurus blochii* | 0.000 | 0.071 | 0.266 | | 0.949 |
| *Acanthurus maculiceps* | 0.000 | 0.071 | 0.266 | | 0.950 |
| *Caranx melampygus* | 0.000 | 0.071 | 0.266 | | 0.951 |
| *Chromis delta* | 0.000 | 0.071 | 0.266 | | 0.952 |
| *Coris gaimard* | 0.000 | 0.071 | 0.266 | | 0.953 |
| *Diagramma pictum* | 0.000 | 0.071 | 0.266 | | 0.954 |
| *Kyphosus pacificus* | 0.000 | 0.071 | 0.266 | | 0.955 |
| *Lutjanus gibbus* | 0.000 | 0.071 | 0.266 | | 0.956 |
| *Pterocaesio marri* | 0.000 | 0.071 | 0.266 | | 0.957 |
| *Rhinecanthus rectangulus* | 0.000 | 0.071 | 0.266 | | 0.958 |
| *Scolopsis affinis* | 0.000 | 0.071 | 0.266 | | 0.959 |
| *Scolopsis lineata* | 0.000 | 0.071 | 0.266 | | 0.960 |
| *Stegastes fasciolatus* | 0.000 | 0.071 | 0.266 | | 0.961 |
| *Anampses geographicus* | 0.000 | 0.071 | 0.268 | | 0.962 |
| *Calotomus japonicus* | 0.000 | 0.071 | 0.268 | | 0.962 |
| *Chaetodon auripes* | 0.000 | 0.071 | 0.268 | | 0.963 |
| *Chromis yamakawai* | 0.000 | 0.071 | 0.268 | | 0.964 |
| *Chrysiptera starcki* | 0.000 | 0.071 | 0.268 | | 0.965 |
| *Macolor niger* | 0.000 | 0.071 | 0.268 | | 0.966 |
| *Mulloidichthys vanicolensis* | 0.000 | 0.071 | 0.268 | | 0.967 |
| *Paracaesio xanthura* | 0.000 | 0.071 | 0.268 | | 0.968 |
| *Plectorhinchus lessonii* | 0.000 | 0.071 | 0.268 | | 0.969 |
| *Pseudojuloides elongatus* | 0.000 | 0.071 | 0.268 | | 0.969 |
| *Scarus fuscocaudalis* | 0.000 | 0.071 | 0.268 | | 0.970 |
| *Bodianus axillaris* | 0.071 | 0.000 | 0.273 | | 0.971 |
| *Chromis vanderbilti* | 0.071 | 0.000 | 0.273 | | 0.972 |
| *Ostracion meleagris* | 0.071 | 0.000 | 0.273 | | 0.973 |
| *Parupeneus crassilabris* | 0.071 | 0.000 | 0.273 | | 0.974 |
| *Sargocentron spiniferum* | 0.071 | 0.000 | 0.273 | | 0.975 |
| *Stethojulis interrupta* | 0.071 | 0.000 | 0.273 | | 0.975 |
| *Amphiprion ocellaris* | 0.071 | 0.000 | 0.273 | | 0.976 |
| *Cromileptes altivelis* | 0.071 | 0.000 | 0.273 | | 0.977 |
| *Dascyllus reticulatus* | 0.071 | 0.000 | 0.273 | | 0.978 |
| *Elagatis bipinnulata* | 0.071 | 0.000 | 0.273 | | 0.979 |
| *Heniochus singularius* | 0.071 | 0.000 | 0.273 | | 0.980 |
| *Labracinus* sp. | 0.071 | 0.000 | 0.273 | | 0.980 |
| *Lethrinus nebulosus* | 0.071 | 0.000 | 0.273 | | 0.981 |
| *Pomacanthus imperator* | 0.071 | 0.000 | 0.273 | | 0.982 |
| *Pomacentrus* sp. | 0.071 | 0.000 | 0.273 | | 0.983 |
| *Pomachromis* sp. | 0.071 | 0.000 | 0.273 | | 0.984 |
| *Syphraena* sp. | 0.071 | 0.000 | 0.273 | | 0.984 |
| *Trimma caudomaculatum* | 0.071 | 0.000 | 0.273 | | 0.985 |
| *Centropyge bispinosa* | 0.000 | 0.071 | 0.270 | | 0.986 |
| *Cheilodipterus intermedius* | 0.000 | 0.071 | 0.270 | | 0.987 |
| *Chrysiptera unimaculata* | 0.000 | 0.071 | 0.270 | | 0.988 |
| *Heniochus varius* | 0.000 | 0.071 | 0.270 | | 0.988 |
| *Lutjanus bohar* | 0.000 | 0.071 | 0.270 | | 0.989 |
| *Myripristis hexagona* | 0.000 | 0.071 | 0.270 | | 0.990 |
| *Scarus globiceps* | 0.000 | 0.071 | 0.270 | | 0.991 |
| *Scarus rubroviolaceus* | 0.000 | 0.071 | 0.270 | | 0.991 |
| *Atrosalarias holomelas* | 0.071 | 0.000 | 0.273 | | 0.992 |
| *Cheiloprion labiatus* | 0.071 | 0.000 | 0.273 | | 0.993 |
| *Parupeneus indicus* | 0.071 | 0.000 | 0.273 | | 0.994 |
| *Thalassoma trilobatum* | 0.071 | 0.000 | 0.273 | | 0.995 |
| *Amphiprion sandaracinos* | 0.071 | 0.000 | 0.274 | | 0.995 |
| *Centropyge bicolor* | 0.071 | 0.000 | 0.274 | | 0.996 |
| *Cephalopholis urodeta* | 0.071 | 0.000 | 0.274 | | 0.997 |
| *Chromis* sp. | 0.071 | 0.000 | 0.274 | | 0.997 |
| *Chromis weberi* | 0.071 | 0.000 | 0.274 | | 0.998 |
| *Cirrhitichthys aprinus* | 0.071 | 0.000 | 0.274 | | 0.999 |
| *Pterocaesio tile* | 0.071 | 0.000 | 0.274 | | 0.999 |
| *Thalassoma amblycephalum* | 0.071 | 0.000 | 0.274 | | 1.000 |
